# Multi-layered control of Galectin-8 mediated autophagy during adenovirus cell entry through a conserved PPxY motif in the viral capsid

**DOI:** 10.1371/journal.ppat.1006217

**Published:** 2017-02-13

**Authors:** Charlotte Montespan, Shauna A. Marvin, Sisley Austin, Andrew M. Burrage, Benoit Roger, Fabienne Rayne, Muriel Faure, Edward M. Campell, Carola Schneider, Rudolph Reimer, Kay Grünewald, Christopher M. Wiethoff, Harald Wodrich

**Affiliations:** 1 MFP CNRS UMR 5234, Microbiologie Fondamentale et Pathogénicité, Université de Bordeaux, Bordeaux, France; 2 Department of Microbiology and Immunology, Loyola University Chicago Stritch School of Medicine, Maywood, Illinois, United States of America; 3 Heinrich-Pette-Institut, Leibniz-Institut für Experimentelle Virologie, Hamburg, Germany; Stony Brook University, UNITED STATES

## Abstract

Cells employ active measures to restrict infection by pathogens, even prior to responses from the innate and humoral immune defenses. In this context selective autophagy is activated upon pathogen induced membrane rupture to sequester and deliver membrane fragments and their pathogen contents for lysosomal degradation. Adenoviruses, which breach the endosome upon entry, escape this fate by penetrating into the cytosol prior to autophagosome sequestration of the ruptured endosome. We show that virus induced membrane damage is recognized through Galectin-8 and sequesters the autophagy receptors NDP52 and p62. We further show that a conserved PPxY motif in the viral membrane lytic protein VI is critical for efficient viral evasion of autophagic sequestration after endosomal lysis. Comparing the wildtype with a PPxY-mutant virus we show that depletion of Galectin-8 or suppression of autophagy in ATG5-/- MEFs rescues infectivity of the PPxY-mutant virus while depletion of the autophagy receptors NDP52, p62 has only minor effects. Furthermore we show that wildtype viruses exploit the autophagic machinery for efficient nuclear genome delivery and control autophagosome formation via the cellular ubiquitin ligase Nedd4.2 resulting in reduced antigenic presentation. Our data thus demonstrate that a short PPxY-peptide motif in the adenoviral capsid permits multi-layered viral control of autophagic processes during entry.

## Author summary

Cells have developed surveillance systems to detect invading pathogens, e.g. when they damage the membrane upon cell entry. Cells respond to membrane damage by activating selective autophagy to target pathogens for lysosomal degradation and pathogen removal. In this manuscript we show that endosome penetrating adenoviruses also activate an autophagic response upon entry and that this involves Galectin-8 mediated membrane damage recognition similar to pathogenic bacteria. In contrast adenoviruses avoid degradation by escaping into the cytosol. We show that they possess a small virion encoded PPxY-peptide motif, which they use to divert the autophagic response of the cell. This motif sequesters the cellular ubiquitin ligase Nedd4.2 resulting in limited autophagosome formation, which prevents viral degradation and antigenic presentation and ensures efficient endosomal escape and nuclear transport.

## Introduction

Intracellular pathogens, such as viruses, penetrate the limiting membrane of the cell to access cellular functions for propagation support. In response, cells try to detect and eliminate entering viruses through multiple pre-existing defense mechanisms referred to as restriction factors or intrinsic immunity [1]. To establish productive infections, viruses thus have to divert, limit or control cellular intrinsic immunity. Adenoviruses (AdV) are amongst the most efficient viruses to enter cells and rapidly establish lytic infections after nuclear genome delivery. AdV are non-enveloped, double stranded DNA viruses, which enter cells by receptor-mediated endocytosis [2,3]. Uptake invokes structural changes in the capsid [4], which releases the membrane lytic internal capsid protein VI (PVI) to breach the endosomal membrane [5,6]. Following membrane rupture, AdVs escape to the cytosol and use microtubule dependent transport accessing the nucleus via the microtubule organizing center (MTOC) [7]. At the nucleus the capsid binds and disassembles at the nuclear pore complex followed by genome import into the nucleus [8].

Membrane penetration is an essential step in the infection process. This was shown in work using the temperature sensitive AdV mutant *ts1* (TS1), which has a point mutation (P137L) in the viral protease gene preventing newly assembled virions from undergoing maturation cleavage at the non-permissive temperature. TS1 particles are hyper stable and enter cells via endocytosis but fail to release PVI. Subsequent absence of membrane penetration results in *ts1* particles being sorted into lysosomes for degradation [9,10]. A key role in AdV cell entry is played by a highly conserved PPxY peptide motif (where x can be any amino acid) in PVI, which is exposed upon PVI release [11]. PPxY motifs bind to WW domains commonly found e.g. in the Nedd4 family of HECT-domain E3 ubiquitin ligases. Using recombinant proteins it was recently shown that PVI binds directly via the PPxY motif to the ubiquitin ligase Nedd4.2 [12]. Mutating the motif to PGAA impairs Nedd4.2 binding and abolishes PVI ubiquitylation. More significantly, although mutation of the PPxY motif does not decrease membrane rupture, for unknown reasons PVI-mutated viruses (M1) have a strong nuclear transport defect and fail to localize at the MTOC, resulting in an up to twenty-fold defect in specific infectivity compared to the wild type (WT) [11].

Membrane damage, caused by viruses or other pathogens, is perceived as a danger signal by the cell and recognized through the galectin system. Galectins are beta-galactoside-binding proteins that contain carbohydrate recognition domains, which sense and mark membrane damage through the abnormal cytosolic exposure of intra-lumenal glycans [13]. Several galectins including Gal3, Gal9 and Gal8 mark bacteria induced membrane damage although only Gal8 was shown to restrict bacterial replication [14,15]. Galectin positive membrane fragments can be subject to degradation via autophagy [15,16]. This is achieved e.g. through Gal8 mediated recruitment of the adapter protein NDP52 towards bacteria containing vacuoles. NDP52 in turn establishes the link to the autophagic machinery for cargo degradation exemplifying how the initial sensing via galectins leads to an active antimicrobial response [15,17].

Autophagy, or selective autophagy when the cargo is sequestered via receptors, is an evolutionary-conserved cytosolic, lysosome-dependent, degradation process [18]. It serves as a cellular survival pathway to maintain homeostasis e.g. by providing the cell with nutrients upon starvation. Autophagy is initiated through formation of an isolated membrane structure, the phagophore, via the concerted action of several factors belonging to the AuTophaGy-related genes (ATG) [19,20]. After initiation the cytoplasmic protein LC3 (Microtubule-associated protein 1A/1B-light chain 3) becomes lipidated and incorporated into the elongating membrane, which forms a double-membrane vesicular structure, the autophagosome, and closes around the cargo destined for degradation. In contrast autophagosome formation and maturation into autolysosomes is still not fully understood but may involve LC3 mediated transport from the site of origin towards the perinuclear region by using a retrograde, microtubule dependent and dynein-dynactin motor complex mediated transport. This active transport facilitates efficient fusion of closed autophagosomes with perinuclear lysosomes [21–23].

Selective autophagy is part of the intrinsic immune response to attenuate intracellular pathogen infections via lysosomal degradation [24]. Next to galectins, selective autophagy involves additional marker molecules such as ubiquitin and further adapter molecules [25]. We recently demonstrated that Gal3 also marks membrane damage caused by AdV and observed that AdV traffics in Gal3 positive membranes prior to endosomal escape [5,26] although a role in selective autophagy was not addressed. Still, several studies have reported that autophagy can promote or restrict viral infections including AdV [27–29]. Autophagy also indirectly restricts viral infection by increasing viral peptide presentation on MHC-II molecules to mount an adaptive immune response as recently shown for AdV [28] and other viruses [30,31]. Accordingly several viruses have developed efficient strategies to temper with the autophagic machinery [32].

Within this study we use AdV to show that selective autophagy mediated by Gal8 also targets endosomolytic viruses. Moreover we show that AdV escape from and limit the autophagic response through a conserved PPxY peptide motif encoded in the membrane lytic capsid protein VI. We show that this is achieved by restricting the formation of autolysosomes via a mechanism involving the cellular ubiquitin ligase Nedd4.2 resulting in reduced antigenic presentation. Furthermore we provide evidence that AdV hijacks the autophagic response to achieve efficient endosomal escape and accelerated nuclear transport and genome delivery showing a multilayered viral control of the autophagic machinery during virus entry.

## Results

### Microtubules and a conserved PPxY motif in capsid protein VI are required for AdV efficient endosomal escape

**Fig 1:**
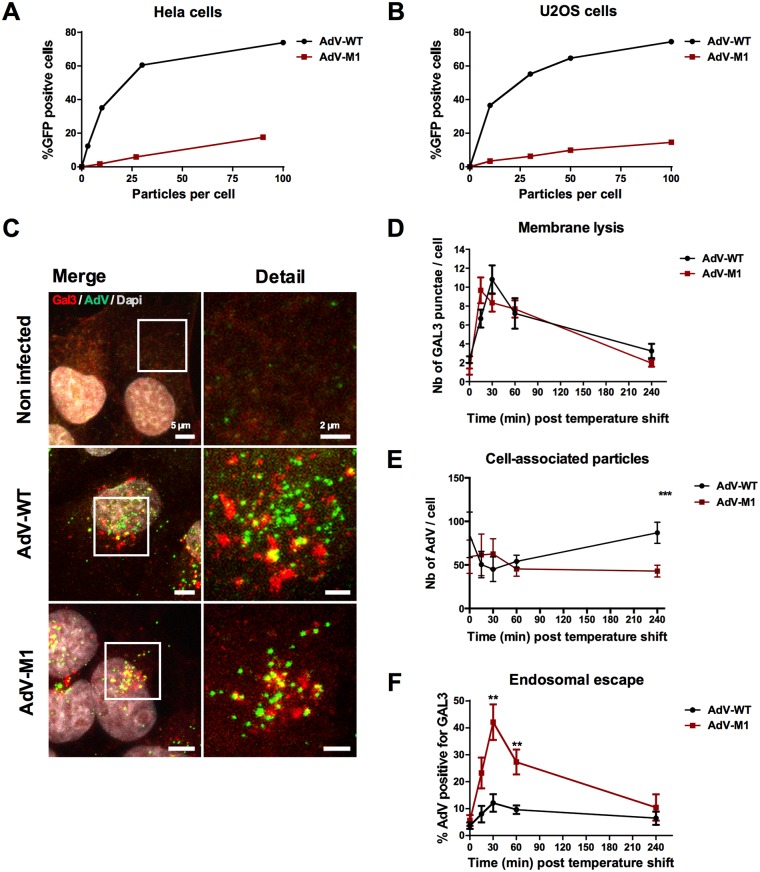
AdV endosomal escape is PPxY dependent. HeLa cells (A) or U2OS cells (B) were infected with varying amounts of adenoviral vector particles expressing GFP (WT in black, M1 in red) and the percentage of GFP positive cells was determined 24hpi by FACS analysis. (C) U2OS cells expressing Gal3-mCherry (red signal) were infected with WT or M1 viruses and fixed at 30 minutes post infection and stained for AdV (green signal). Note that colocalization (yellow signal) indicates membrane rupture. (D) U2OS cells were infected with fluorescent viruses fixed at different time points and stained for endogenous GAL3. At indicated time points Gal3 positive signals where quantified. The total number of Gal3 punctae is indicated. (E) U2OS cells were infected as in D and cell-associated virus was quantified at indicated time points. (F) Colocalization between Gal3 and AdV signals was quantified and is displayed as percentage colocalization of total AdV signal. **: P<0.01 and errors bars are standard error. (See also S1 Fig).

**Fig 2:**
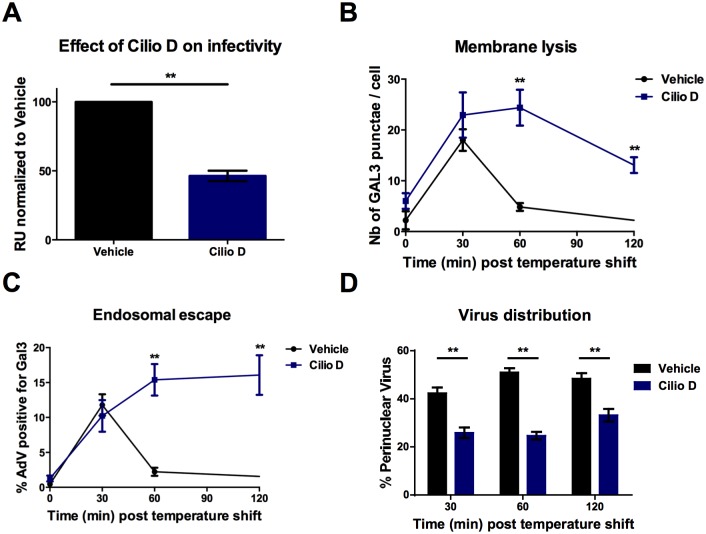
AdV endosomal escape is dynein dependent. (A) Cells were pretreated with 100 μM of Ciliobrevin D for 30 minutes or DMSO and transduced with GFP expressing WT vector in presence of drugs. Twenty four hours later transduction levels were determined by FACS and normalized for the vehicle control (B) Cells were infected with fluorescent viruses in presence and absence of 100μM CilioD and fixed at different time points. The number of Gal3 punctae per cell was determined (C) Cells were infected as in B. Colocalization between Gal3 and AdV signals was quantified and is displayed as percentage of colocalization. (D) Cells were infected as in B. Fluorescent viruses in control or drug treated or control cells were quantified at indicated time points in the perinuclear region vs. the rest of the cytosol. Error bars (SE) show cell-to-cell variations.

We showed previously that E1/E3 deleted GFP expressing adenoviral vectors with the PPxY-motif (WT) in capsid protein VI mutated to PGAA (M1) have reduced transduction efficiency and altered nuclear transport [11]. Both vectors were used throughout this study and are referred to as “WT” and “M1” respectively. To verify the phenotype we infected cells with WT and M1 and quantified the transduction levels at different multiplicities of infection (MOI). The results confirmed the M1 phenotype in a variety of cell types suggesting a virion-associated mechanism (Figs 1A, 1B and S1). We then asked if the defect was linked to the role of PVI in membrane penetration. We infected cells for different times with WT and M1 and used Gal3 as a marker for AdV induced membrane damage. The results showed that both viruses efficiently induce Gal3 punctae (Fig 1C). We quantified both the number of Gal3 punctae per cell as a measure of membrane rupture and the colocalization of virus particles with Gal3 as a measure for endosomal escape. We observed no apparent difference in the kinetics of membrane lysis between WT and M1 (Fig 1D) and only minor differences in the absolute number of cell-associated virus over time (Fig 1E). In contrast, the M1 colocalized with Gal3 punctae to a much larger extend than WT (Fig 1F), suggesting a post-lysis endosomal escape defect. Previous *in vivo* imaging of AdV endosomal escape suggested a propelled and microtubule dependent escape mechanism [5,26] and both dynein and microtubule transport have been implicated in AdV entry [33,34]. To confirm that AdV utilize dynein to escape from ruptured endosomes we used Ciliobrevin D (CilioD), a reversible chemical inhibitor of the dynein AAA+ ATPase motor to inhibit dynein activity during viral transduction (Fig 2). At non-toxic CilioD concentrations we observed decreased GFP transgene expression compared to vehicle control treated cells (Fig 2A). Time course infection experiments with fluorescently labeled WT revealed no change in the initial number of Gal3 punctae upon CilioD treatment. However, Gal3 punctae increased over time (Fig 2B) and the WT remained associated with Gal3 (Fig 2C) in drug-treated cells suggesting involvement of dynein in virus escape and membrane damage removal but not in the initial lysis. Sequestration of the WT after CilioD treatment also prevented efficient virus translocation to the perinuclear area (Fig 2D) consistent with previous observations made for the M1 virus [11]. Taken together, these data show that the PVI PPxY-motif and dynein are critical for efficient escape from ruptured endosomes and subsequent transport to the nucleus.

### Adenovirus induces selective autophagy upon endosomal membrane penetration

**Fig 3:**
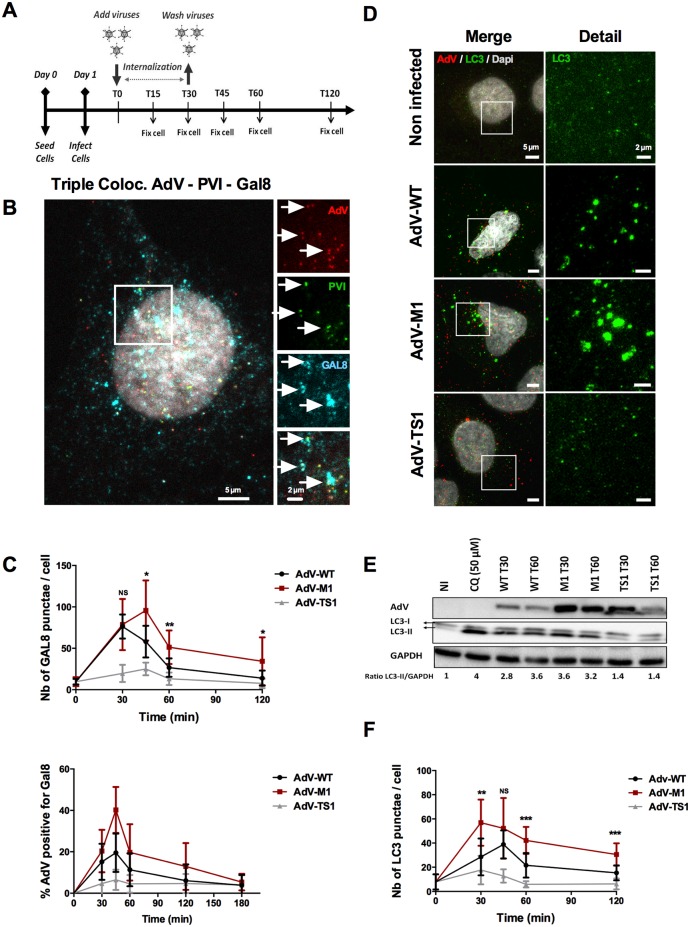
AdV induced membrane damage causes selective autophagy. (A) Schematic representation of the experimental setup for quantitative immune fluorescence analysis. Cells grown on coverslips were infected with virus at 37°C followed by inoculum removal after 30min. Coverslips were collected and fixed at indicated time points for quantitative immunofluorescence analysis as detailed in SI. (B) U2OS cells were infected with WT fluorescently labeled viruses (red signal) and fixed at 30min post infection and stained with anti PVI antibodies (green signal) and anti Gal8 antibodies (cyan signal). The insets to the right show triple colocalization (AdV+VI+Gal8) indicated by arrows (white signal). (C) U2OS cells were infected with fluorescently labeled WT (black line), M1 (red line) or TS1 viruses (gray line) as outlined in (A). At each time points cells were fixed and stained for Gal8. The top panel shows the absolute number of Gal8 punctae per cell, the bottom panel the percentage of Gal8 positive particles. Error bars show cell-to-cell variations (n>10 cells; NS: no significant; *: P<0.05; **: P<0.01; ***: P<0.005 and errors bars are standard deviation). (D) Representative confocal images of cells infected with different AdV showing virus induced autophagy. Conditions are indicated to the left of each row. Cells were fixed at 30 minutes post infection and stained with anti-AdV (first column, red signal) and anti-LC3 (first and second columns, green signal). (E) Infected U2OS cells were analyzed by western blot at 30 min (T30) and 60 min (T60) post infection using LC3 and GAPDH specific antibodies. As a positive control cells were treated with 50μM of CQ during 3hours. The LC3 signal depicts unconjugated (LC3-I) or PE-conjugated (LC3-II) LC3. Note that upon PE conjugation LC3 has a higher mobility in SDS-PAGE. The ratio of LC3II/GAPDH normalized to the non-infected condition was determined and is given below the panel. (F) Experiment as in (C) stained with anti-LC3 depicting the absolute number of LC3 punctae per cell.

**Fig 4:**
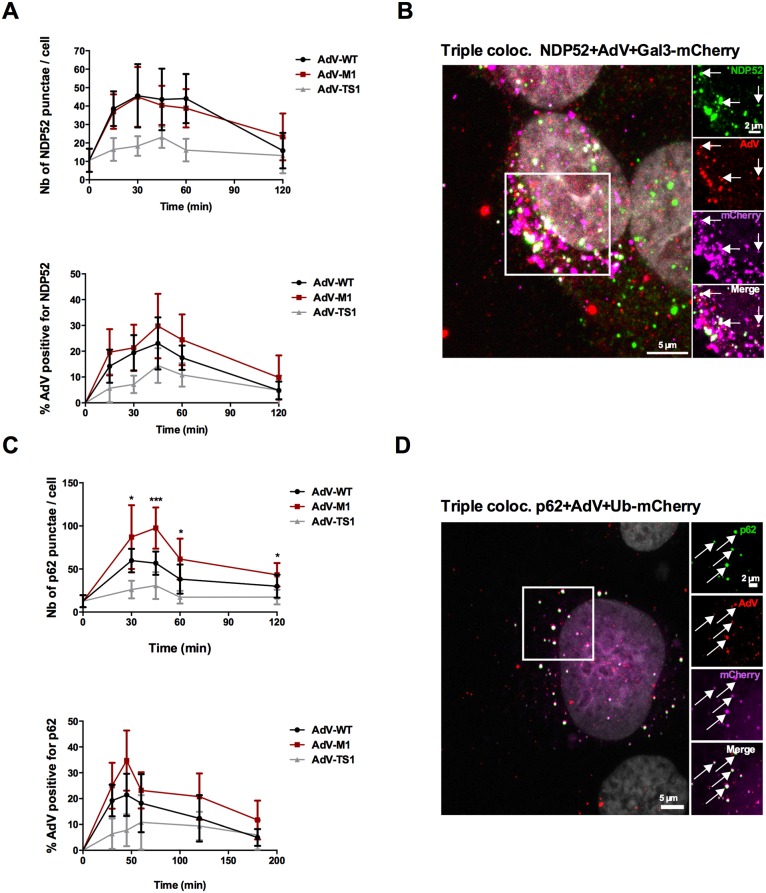
Recruitment of adaptor proteins NDP52 and p62 upon entry. (A) U2OS cells were infected with WT (black line), M1 (red line) or TS1 viruses (gray line) as outlined in Fig 3A. At each time points cells were fixed and stained for NDP52. The top panel shows the absolute number of NDP52 punctae per cell, the bottom panel the percentage of NDP52 positive particles. Error bars show cell-to-cell variations (n>10 cells; NS: no significant; *: P<0.05; **: P<0.01; ***: P<0.005 and errors bars are standard deviation). (B) U2OS cells stably expressing Gal3-mCherry (depicted as magenta signal) were infected with WT viruses and fixed at 30min post infection and stained with anti-AdV (red signal) and anti-NDP52 (green signal). The insets to the right show triple colocalization (AdV+Gal3mCh+NDP52) indicated by arrows (white signal). (C) U2OS cells were infected as in (A) and stained for p62. The top panel shows is the absolute number of p62 punctae per cell, the bottom panel the percentage of p62 positive particles. (D) U2OS cells transfected with a plasmid encoding Ubiquitin-mCherry (depicted as magenta signal) were infected with WT viruses and fixed at 30min post infection and stained with anti-AdV (red signal) and anti-p62 (green signal). The insets to the right show triple colocalization (AdV+UbmCh+p62) indicated by arrows (white signal).

We next asked if AdV, similar to invasive bacteria, would activate selective autophagy upon membrane rupture. To address this question we used three different AdV’s all based on genotype HAdV-C5, which share receptor-mediated endocytosis but differ upon endosomal sorting. Next to WT and M1 we infected cells with an E1/E3 deleted GFP expressing *ts1* adenoviral vector grown at the non-permissive temperature [10]. This hyperstable *ts1* mutant virus (“TS1” in this study) does not release PVI and does not lyse the endosome upon uptake. We then performed an infection time course (shown in Fig 3A) and quantified appearance of Gal8 punctae to mark membrane damage. We used Gal8 because of its functional role in selective autophagy [15]. Gal8 punctae formation occurred in WT and M1 but not TS1 infected cells (S2 Fig), was often linked to particles also positive for PVI (Fig 3B) and punctae formation and particle association was rapid (Fig 3C) and followed similar kinetics as Gal3 punctae formation. To show that AdV induced membrane damage activates autophagy we next stained for LC3 and quantified LC3 punctae reminiscent of autophagosome formation [35]. We observed LC3 punctae formation for WT and M1 but not for TS1 supporting that membrane lysis was required to initiate autophagy (Fig 3C). We confirmed the results in a different cell type (S2B Fig) and by showing the membrane damage-dependent accumulation of phosphatidyl-ethanolamin (PE) conjugated LC3-II by western blot in WT and M1 but not in TS1 infected cells (Fig 3E). LC3 punctae formation occurred rapidly within 15–30 min after virus uptake (Fig 3F) resembling PVI release kinetics [10]. We then asked if AdV induced autophagy was selective and mediated by adapter proteins. We repeated the time course experiment and analyzed recruitment of autophagy adapter p62, NDP52 and optineurin. Accumulation of p62 and NDP52 punctae was dependent on virus induced membrane damage (S2C and S2D Fig). Punctae appearance and association with WT and M1 particles followed similar kinetics (Fig 4A for NDP52 and 4C for p62) as previously observed for galectins and LC3. In contrast optineurin, was not detected in association with viral particles under our conditions. Co-staining for cellular marker molecules and viruses showed large overlap in colocalization suggesting functional links. E.g. several viruses positive for NDP52 also stained positive for Galectins (Fig 4B) and p62 positive viruses stained also positive for ubiquitin (Fig 4D) reminiscent of their recruitment towards invasive bacteria.

In summary our analysis suggested that PVI released from entering viral particles caused membrane damage to which the cell responds with selective autophagy. Interestingly we observed higher levels and more prolonged accumulation of LC3 punctae for the escape defective mutant M1 vs. WT (Fig 4F). We hypothesized that M1 virions maybe degraded by autophagy, which could explain their strongly reduced infectivity.

### AdV with mutated PPxY motif in capsid protein VI are subject to autophagic degradation

**Fig 5:**
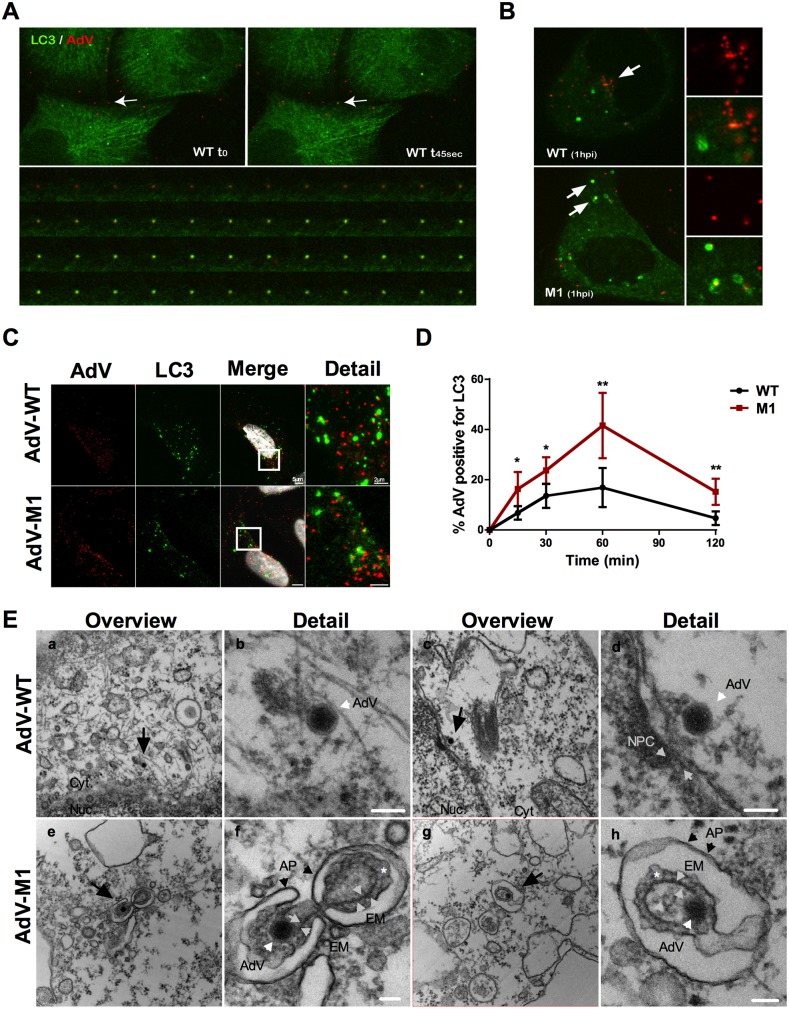
The PPxY-mutant M1 associates with autophagosomes. (A) Live-cell imaging showing LC3 acquisition of WT upon entry. Stable expressing U2OS-LC3-GFP cells were infected with Alexa594 coupled WT and imaged using spinning-disk confocal microscopy. The top two images show individual frames separated by ~45 seconds from S1 Movie. The arrow points to an LC3 negative virus (left panel) becoming LC3-positive (right panel). The bottom panel shows a higher magnification and frame resolution of the same event. (See also S1 Movie). (B) The panel shows single frames of cells infected as in (A) either with WT (top) or M1 virus (bottom) at 1 hpi. The arrow at the top panel points to the microtubule organizing center where WT viruses accumulate (shown at higher magnification to the right). The arrows at the bottom panel point to autophagosomes engulfing mutant M1 viruses (shown at higher magnification to the right). (C) Representative confocal images of cells at 1hpi infected with WT (top row) and M1 (bottom row) and stained for AdV (red signal) and LC3 (green signal). Virus association with LC3 appears as yellow signal (see detail). (D) Experiment as in (C). The percentage of each AdV colocalizing with LC3 was quantified over time and is given as percentage of total virus. Error bars show cell to cell variation (n>10 cells; *: P<0.05; **: P<0.01). (E) TEM analysis of U2OS cells infected for 30 minutes with WT (a-d, top row) or M1 (e-h, bottom row). The overview images show cytosolic WT viruses (a, c) and vesicle associated M1 viruses (e, g) depicted by a black arrow. At higher magnification WT (b, d) and M1 (f, h) particles (AdV) are depicted by white arrowheads. The nuclear pore complex (NPC in d) and the endosomal membrane (EM in f, g) are indicated with grey arrowheads and autophagosomes (AP) by black arrowheads (f, h). The * indicates the putative location for autophagy receptors between EM and AP. Error bars are 100 nm.

To determine if M1 viruses are degraded via autophagy we first visualized AdV association with LC3 in living cells. We infected cells stably expressing LC3-GFP [36] with Alexa594 coupled WT and M1 virions and imaged the cells using spinning disk confocal microscopy. We observed that within ~15–30 min several cell associated viruses turned LC3 positive, mostly occurring at the cellular periphery (Fig 3A, S1 Movie). At >1hpi most WT viruses showed no more association with LC3 and accumulated in the vicinity of the nucleus (Fig 3B and 3C, top). In contrast, M1 viruses lacked nuclear accumulation and were often found inside large LC3 positive structures reminiscent of autophagosomes (Fig 3B and 3C, bottom). This difference in dynamic LC3 association between WT and M1 viruses was confirmed using quantitative time resolved fluorescence analysis (Fig 3D). Autophagosomes are characterized by forming a double membrane around their substrate. To confirm that M1 particles associate with autophagosomes we next performed transmission electron microscopy (TEM). Cells were infected with fluorescently labeled WT or M1 virus, fixed at 30 min post infection and processed as detailed in the material and methods section. WT viruses were observed in the cytoplasm (Fig 5Ea and 5Eb) or at the nuclear pore complex (NPC, Fig 5Ec and 5Ed). In contrast M1 viruses could be observed inside vesicular structures (Fig 5Ee and 5Eg). At higher magnification we could observe that M1 particles seemed to be entrapped in partially ruptured vesicles resembling endosomes (Fig 5Ef and 5Eh). These virus containing vesicles were partially (f) or fully (h) engulfed by a double membrane vesicle resembling autophagosomes. Interestingly we observe a cleft between the endosome and the inner autophagosomal membrane filled with electron dense material, which would be the putative location for the linking autophagy receptors (marked with asterisk in Fig 5Ef and 5Eh). Our analysis thus strongly supports that M1 particles still inside the ruptured vesicle are sequestered by autophagy whereas WT viruses escape from the endosomal compartment prior to autophagic sequestration.

**Fig 6:**
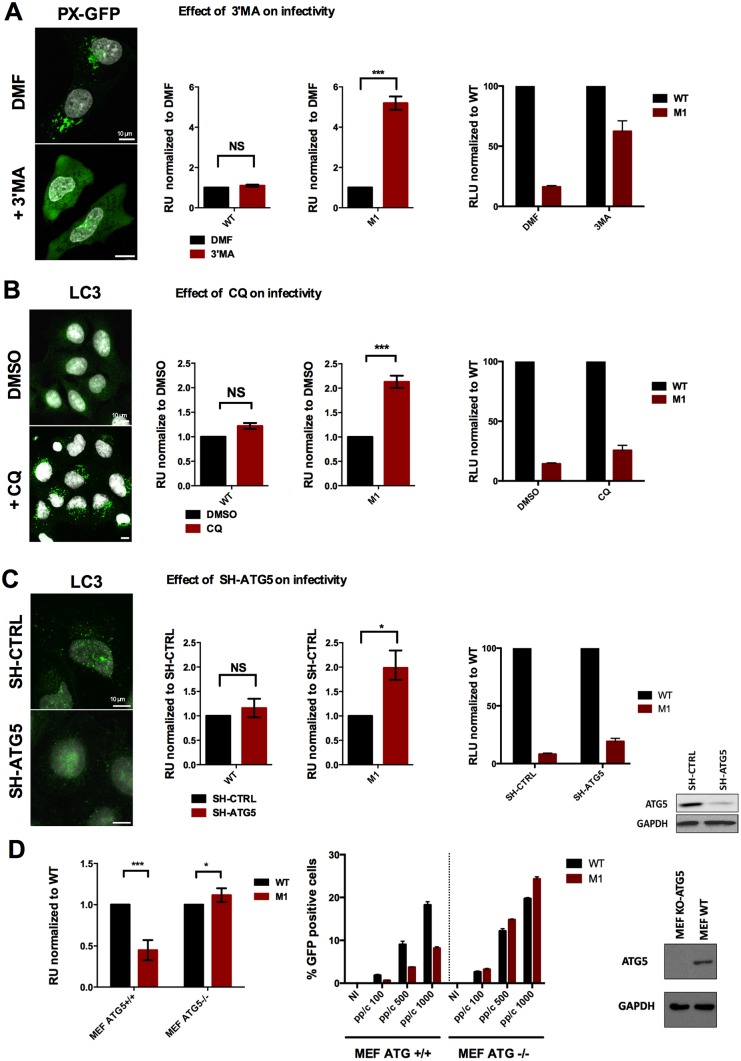
PPxY-mediated endosomal escape prevents autophagic degradation of incoming virions. (A) Left panel: U2OS cells expressing the Pi3P *in cellulo* binding probe PX-GFP were treated with vehicle (top) or with 5mM of the Pi3K inhibitor 3’MA (bottom). Middle panel: U2OS cells pre-treated with vehicle alone (black bars) or 3’MA (red bars) were transduced with WT or M1. Transgene expression was determined and normalized to vehicle treated controls to show the fold induction of infectivity upon treatment. Right panel: The same data as in the middle panel showing the level of M1 infectivity rescue compared to the normalized WT infectivity upon treatment. (B) Left panel: U2OS cells were treated with vehicle (top) or with chloroquine (CQ, 50μM, bottom) to block the autophagic flux, fixed and stained for LC3. Middle panel: Cells were treated with vehicle alone (black bars) or chloroquine (red bars) and transduced with WT or M1. Transgene expression was determined and normalized to vehicle treated controls to show the fold induction of infectivity. Right panel: The same data as in the middle panel showing the level of M1 infectivity rescue compared to normalized WT infectivity. (C) Left panel: U2OS cells depleted for ATG5 (SH-ATG5) or control depleted cells (SH-CTRL) and starved using HBSS during 4h, fixed and stained for LC3. Middle panel: Cells were transduced with WT or M1 and the relative transduction efficiency in SH-CTRL cells (black bars) and SH-ATG5 cells (red bars) was determined. Right panel: The same data as in the middle panel showing the level of M1 infectivity rescue compared to normalized WT infectivity. ATG5 expression levels were determined by western blot. (D) Left panel: Control MEFs (ATG5 +/+) and KO MEFs (ATG5 -/-) were transduced with WT or M1 as indicated and the relative transduction efficiency for the M1 (red bars) compared to the WT (black bars) was determined. Right panel: The panel shows the absolute number of transduced cells at indicated amounts of physical particles added to the cell (pp/c) for the WT (black bars) and the M1 (red bars) in ATG5 control (left) and KO (right) MEFs. ATG5 expression levels were determined by western blot.

To investigate a potential autophagic degradation for the M1 virus we treated cells with 3-methyladenin (3-MA), which blocks autophagosome formation via the inhibition of class III PI3K [35]. Pretreated and vehicle control cells were transduced with WT or M1 viral vector expressing GFP and relative transduction levels were determined using fluorescence activated cell sorting (FACS). 3-MA treatment did not affect WT virus infectivity but significantly increased M1 virus infectivity restoring WT infectivity to nearly 75% (Fig 6A). In addition we found that the M1 virus located *per se* more than the WT virus with Pi3P positive compartments from which autophagosome formation originates (S3A Fig) [37] and that upon M1 infection Beclin-1, a subunit of the class III PI3K kinase complex, was enriched on cellular membranes (S3B and S3C Fig) suggesting differences in class III PI3K kinase complex activity between both viruses. We next inhibited the autophagic flux by blocking lysosome acidification with chloroquine (CQ, Fig 6B) [35]. CQ treatment increased M1 virus infectivity without affecting WT infectivity although the effects were more moderate (Fig 6B). Next we depleted cells of the LC3 conjugation factor ATG5 using SH-RNAs [35]. Again we observed a specific but moderate increase in M1 infectivity (Fig 6C). Because of the reduced impact in restoring M1 infectivity and because our depletion efforts yielded only partial removal of ATG5 we next infected ATG5 -/- MEFs (Mouse Embryonic Fibroblasts) with WT and M1. As shown in Fig 6D absence of ATG5 in MEFs fully restored the M1 infectivity although the M1 defect in the murine model was less pronounced than in human cells. Still this analysis strongly supported that autophagy was responsible for the M1 defect.

**Fig 7:**
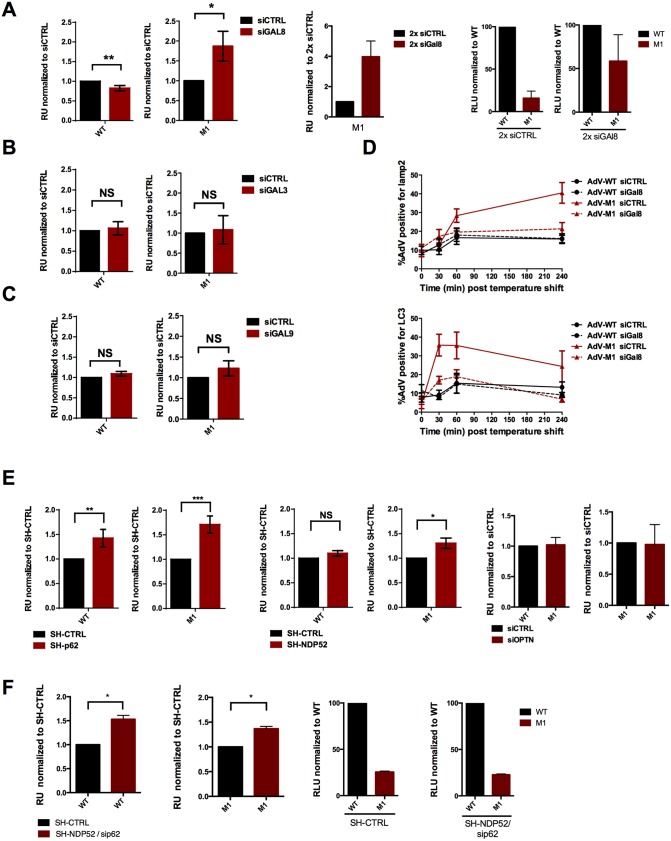
M1 restriction is mediated by Gal8. (A) Left panel: U2OS cells were depleted with siRNAs specific for galectin8. Cells were transduced with WT or M1 as indicated and the relative transduction efficiency was calculated for control depleted cells (black bars) or galectin depleted cells (red bars). Middle panel: Relative transduction efficiency of the M1 virus following two rounds of siRNA depletion. Right panel: The same data as in the middle panel showing the level of M1 infectivity rescue compared to the normalized WT infectivity upon treatment. Each experiment was done in triplicate (B) Experiment essentially done as in (A) except that galectin 3 was depleted. (C) Experiment essentially done as in (A) except that galectin 9 was depleted. (D) HeLa cells were transfected twice with control or galectin8 specific siRNAs followed by infection with WT or M1 viruses and fixed at different time points after infection. Quantification of AdV colocalizing with LC3 (top panel.) and Lamp1 (bottom panel) was performed and is shown as percentage of colocalization for each virus and condition according to the legend. (E) Left panel: Cells were depleted with SH-RNA specific for p62. Specific or control depleted cells were transduced with WT or M1 as indicated and the relative transduction efficiency was calculated for control depleted cells (black bars) or p62 depleted cells (red bars). Middle panel: Experiment essentially done as for p62 except that SH-RNA was directed against NDP52. Right panel: Experiment essentially done as for p62 except that SH-RNA was directed against optineurin (OPTN). (F) Left panel: Cells were depleted with SH-RNA specific for NDP52 followed by transfection with siRNA against p62. Double depleted or control depleted cells were transduced with WT or M1 as indicated and the relative transduction efficiency was calculated for control depleted cells (black bars) or p62 depleted cells (red bars). Middle panel: The same data as in the left panel showing the level of M1 infectivity rescue compared to the normalized WT infectivity upon treatment.

Together our results showed that M1 virus infectivity could be fully or partially restored by blocking autophagy at different steps, showing that the M1 virus but not the WT virus becomes an autophagic substrate. To exclude that the increase in M1 infectivity was not due to pleiotropic effects of autophagy inhibition we depleted cells of galectins or adapter molecules that link the virus induced membrane damage to the autophagic machinery (S4A Fig). M1 infectivity increased only after depleting Gal8 (Fig 7A) but not when depleting either Gal3 (Fig 7B) or Gal9 (Fig 7C). The rescue of M1 infectivity was strongly correlated to the depletion level of Gal8 (Fig 7A) and we were able to observe almost complete restoration of WT infectivity in our best depletion experiments. In contrast M1 infectivity was neither influenced by Gal3 depletion levels nor by Gal9 depletion levels. We next determined WT vs. M1 virus association with LC3 and lysosomes in control- and Gal8-depleted cells over time. Gal8 knockdown had no effect on LC3 colocalization with the WT, but colocalization of the M1 with LC3 decreased to WT levels (Fig 7D, top panel). Additionally, M1 colocalization with the lysosome marker Lamp1 significantly decreased in Gal8 knockdown cells strongly suggesting that Gal8 depletion prevented efficient targeting of the M1 virus for autophagic degradation (Fig 7D, bottom panel). Taken together our results identify Gal8 as an essential restriction factor for the M1 virus and show that Gal8 recruitment towards AdV-ruptured membranes is necessary to target virus-containing membranes for autophagosomal degradation.

We next depleted cells from the autophagy receptors p62, NDP52 and optineurin. We observed no rescue of M1 infectivity when depleting optineurin in agreement with the absence of optineurin recruitment to virus particles. M1 infectivity was only slightly but specifically increased upon depletion of the Gal8 specific autophagy adapter NDP52 (Fig 7E). In contrast, depletion of the autophagy adapter p62 slightly increased infectivity for both viruses implying a somewhat different role in AdV entry (Fig 7E). Thus we next asked if co-depletion of autophagy receptors would have a more pronounced effect and would overcome the M1 restriction. Efficient double depletion of NDP52 and p62 gave similar results as depletion of p62 alone excluding an additive effect (Fig 7F). Taken together this implied that none of the autophagy adapters tested plays a major role in M1 restriction, suggesting that Gal8 mediated restriction of bacteria and adenoviruses work at least in part via different pathways.

### AdV limit autophagy and prevent antigenic presentation

**Fig 8:**
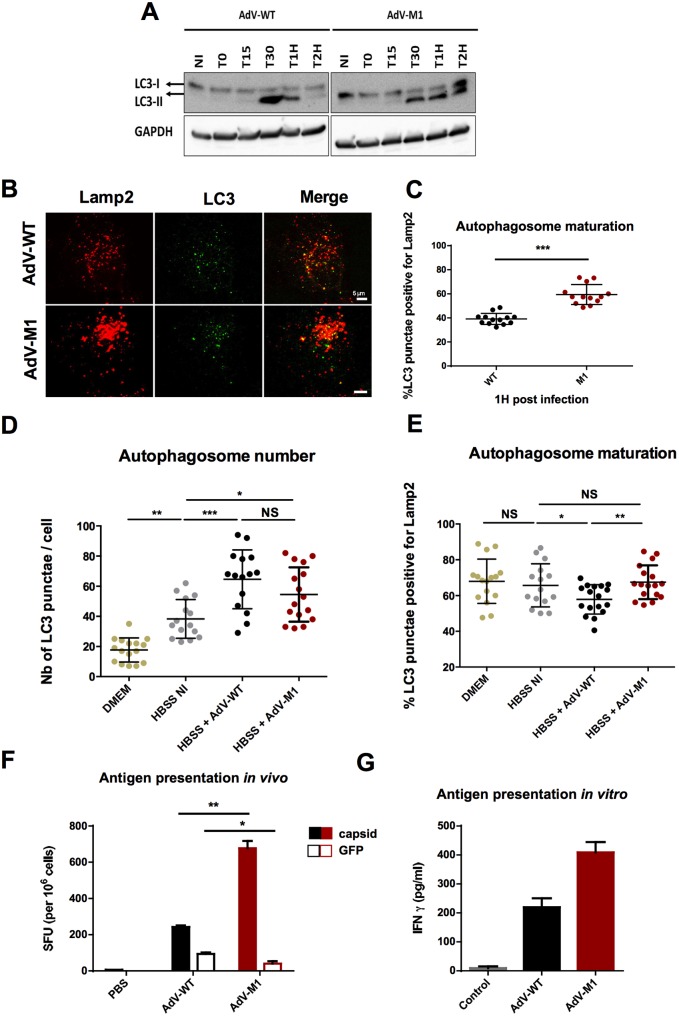
AdV-WT limits autophagosome maturation and antigen presentation. (A) Cells were infected with WT and M1 viruses for indicated time points and cell lysates were analyzed by western blot with LC3 specific antibodies. Specific LC3 bands and GAPDH loading control are indicated. (NI = non infected). (B) The panel shows representative confocal images of U2OS cells infected for 1h with WT or M1 as indicated to left of each row and stained with Lamp2 (red signal) and LC3 (green signal) specific antibodies. (C) Quantification of autolysosomes from the experiment shown in (B) comparing the percentage of LC3 punctae positive for Lamp2 in WT vs. M1 infected cells as indicated below the graph. (D) Cells were starved overnight in HBSS then infected for one hour with AdV as indicated below the graph. Samples were fixed and stained for LC3 and Lamp2. The total number of LC3 punctae per cell at 1hpi is shown. (NI = non infected, DMEM = non starved control cells). (E) Experiment as in (D) showing the percentage of LC3 punctae also positive for Lamp2 (n>13 cells; NS: no significant; *: P<0.05; **: P<0.01; ***: P<0.001). (F) Mice were infected with 10^10^ GFP expressing vector particles of WT, M1 or PBS control. 10 days post infection mice were sacrificed and splenocytes were stimulated with AdV-luc or GFP purified from *E*.*coli*. IFNγ was determined by ELIspot. (G) CD4+ T-cell clones recognizing a conserved AdV hexon epitope were incubated 3:1 with syngeneic APCs transduced with either control media or WT or M1 vectors for 24 hours. IFNγ secretion was quantified by ELISA. (see also S5 Fig).

Autophagy inhibition had little impact on WT infectivity suggesting that AdV might actively target autophagic processes. To address this question we infected cells with WT and M1 and performed a time course western blot analysis to determine the levels of LC3-II. LC3-I conversion initiated within 15–30 min pi for both viruses and continued at least up to 2 hpi for the M1 virus (Fig 8A). In contrast LC3 conjugation induced by the WT virus was comparable in the beginning but declined at ~1hpi and returned to basal levels at 2hpi (Fig 8A). The same experiment performed following CQ treatment, which blocks lysosome acidification and stops the recycling of LC3-II showed LC3-II levels accumulating for the M1 virus but not for the WT virus suggesting that the autophagic flux in WT infected cells was impaired (S5A Fig). We next determined the ratio of autophagosomes to autolysosomes in WT and M1 infected cells at 1hpi. Our analysis showed that at 1hpi ~ 60% of LC3 positive structures in M1 infected cells were also positive for the lysosome marker Lamp2 indicating conversion into autolysosomes. In contrast in WT infected cells only ~40% were Lamp2 positive (Fig 8B and 8C) indicating a defect in autolysosome formation. To confirm that the WT impairs autolysosome formation we transduced cells with LC3 fused to GFP and RFP (LC3-GFP-RFP). Due to the pH-sensitivity of GFP, neutral autophagosomal membranes appear yellow while acidic autophagolysosomes appear red [35]. Quantification in WT and M1 infected cells at 1hpi confirmed a reduced level of autolysosome for the WT (S5B and S5C Fig). To understand how the WT prevents autolysosome formation we first ask if the virus controls the onset of autophagy. We induced non-selective autophagy through overnight starvation followed by infection with WT or M1 and quantified number and maturation state of LC3-positive structures at 1hpi (Fig 8D and 8E). Both viruses induced comparable levels of autophagy marked by an increase in LC3 positive punctae in starved cells (Fig 8D) while the ratio of lamp2 positive vs. lamp2 negative LC3-positive structures was reduced in WT infected cells vs. M1 (Fig 8E) showing that the WT controls autolysosome formation without impairing the onset of autophagy.

Autophagy promotes antigen presentation through fusion with MHC-II containing compartments including antigens incorporated into the AdV capsid [28,31]. Thus we asked if limiting autolysosome formation restricts AdV antigenic presentation. For this we infected mice with equal particle numbers of WT, M1 or PBS control. Ten days later, the mice were sacrificed and purified splenocytes were stimulated with either AdV capsids or GFP purified from *E*.*coli*. T-cell activation was measured by IFNγ ELISPOT (Fig 8F). We saw more IFNγ spot forming units (SFU) in splenocytes stimulated with AdV capsids from the M1 infected mice vs. WT infected mice, suggesting an increased display of AdV capsid antigens during M1 infection. An inverse effect was observed when the splenocytes were stimulated with purified GFP protein and compared to WT infected mice. Because the GFP is only expressed after adenovirus transduction of cells, this observation suggests that there is a defect in M1 infectivity *in vivo*. Additionally, we generated a human CD4^+^ T cell clone that responds to a conserved hexon epitope. We incubated these with syngenic APCs transduced with equivalent viral particles of either WT or M1, and measured T cell activation by IFNγ ELISA (Fig 8G). Again, we saw increased IFNγ levels from the T cells exposed to M1 vs. WT transduced APCs suggesting increased antigen presentation on MHC-II. No significant difference in MHC-II or CD86 expression was observed in human monocyte derived DCs between WT and M1 treated groups, suggesting that differences in the expression of these molecules between treatments did not account for differences in T-cell activation (S5D Fig). Taken together we show that AdV use the PVI PPxY motif to limit capsid epitope presentation on MHC-II presumably by restricting autolysosome formation.

### Nedd4.2 regulates autolysosome formation and AdV nuclear transport

**Fig 9:**
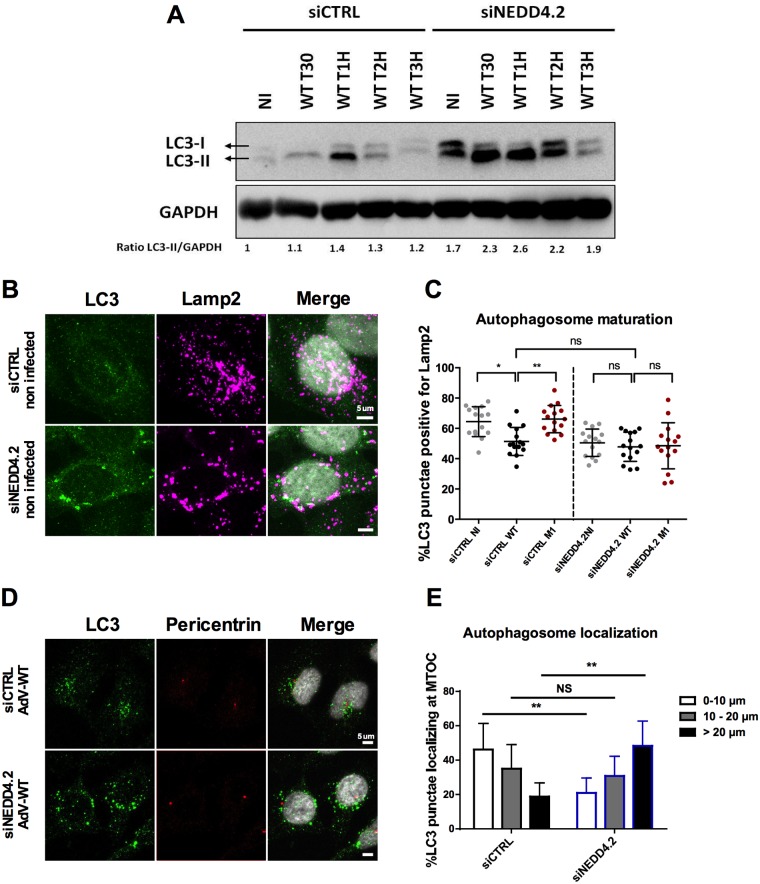
Nedd4.2 controls autophagy upon AdV infection. (A) Nedd4.2 or control depleted cells were infected with WT virus. Cell lysates were analyzed at indicated time points by western blot using LC3 and GAPDH specific antibodies as shown to the left. (NI = non infected). The ratio of LC3II/GAPDH normalized to the non-infected condition in siCTRL depleted cells was determined and is given below the panel. (B) Representative panel of confocal images from Nedd4.2 or control depleted cells (indicated to the left) stained with LC3 and Lamp2 specific antibodies. (C) Nedd4.2 or control depleted cells non-infected (NI) or infected with WT or M1 virus for 1h were fixed and stained as in (B). The percentage of LC3 and Lamp2 positive autolysosomes is indicated for each condition. (n>15 cells; NS: no significant; *: P<0.05; **: P<0.01). (D) Representative panel of confocal images from Nedd4.2 or control depleted cells infected with WT virus for 1h and stained with LC3 and pericentrin specific antibodies. (E) Quantification of the distribution of LC3 dots in cells. Localization was determined by calculating LC3 punctae distribution in concentric circles positioned around pericentrin stain as detailed in SI. The graph shows the color coded relative abundance within the 3 regions. The error bar represents cell-to-cell variation (n>15 cells; NS: no significant; **: P<0.01). (see also S6 Fig).

While several Nedd4 ligases bind the PVI PPxY motif, we previously showed that only depletion of Nedd4.2 caused a transport defect and reduced infectivity [11]. To test if Nedd4.2 was also involved in AdV control of autophagy we infected Nedd4.2 depleted cells (S6A Fig) with WT virus and analyzed LC3-I conversion into LC3-II by western blot (Fig 9A). We observed increased and prolonged levels of LC3-II in infected cells following Nedd4.2 depletion, however, basal levels of LC3-II were also elevated. In individual cells (including non-infected cells) LC3 positive structures appeared more abundant, were larger in size and showed differences in subcellular distribution compared to control cells (Fig 9B and 9D). We next analyzed the percentage of autolysosomes by costaining LC3 with Lamp2 in control vs. Nedd4.2 depleted cells after AdV infection. In control depleted cells the proportion of autolysosomes in WT infected cells was reduced compared to M1 infected or non-infected control cells similar to our previous observation. In contrast upon depletion of Nedd4.2 all cells showed similar reduced levels of autolysosomes including M1 infected and non-infected control cells, without any further reduction in WT infected cells (Fig 9C). These results identified an important physiological role for Nedd4.2 in autolysosome formation and suggested that the WT virus interferes with Nedd4.2 to limit autolysosome formation. We next addressed the physiological role for Nedd4.2 in autophagy regulation in more detail. We analyzed autophagosome formation in Nedd4.2 depleted cells using starvation induced autophagy without viral infection. Under starvation conditions, depletion of Nedd4.2 prevented fusion of LC3 and Lamp2 positive structures (S6B and S6D Fig). The basal level of autophagosomes was higher in Nedd4.2 depleted cells compared to control cells. Starvation increased the number of autophagosomes in both, depleted and control cells (S6C Fig) showing that Nedd4.2 does not prevent autophagy induction. In contrast under both basal and starvation induced autophagy conditions the percentage of autolysosomes was lower in Nedd4.2 depleted cells than in control cells showing that Nedd4.2 is involved in autophagosome maturation into autolysosomes independent of virus infections (S6D Fig). We next investigated the change in subcellular distribution of autophagosomes upon infection. In WT infected cells at 1hpi LC3 positive structures clearly accumulated near the MTOC (Fig 9D) a distribution that is typical for AdV and other incoming viruses [7,38]. Interestingly, upon infection of Nedd4.2 depleted cells LC3 positive structures lost the MTOC accumulation and displayed a more perinuclear distribution (Fig 9D). We confirmed this observation by quantifying the proximity of LC3 positive structures to the MTOC (marked by pericentrin stain) in control and Nedd4.2 depleted cells at 1 hpi (Fig 9E). Because we previously observed a similar MTOC accumulation defect in Nedd4.2 depleted cells with WT viruses [11], we next asked if autophagy contributes to nuclear transport of AdV.

### Autophagy is required for efficient nuclear transport of AdV

**Fig 10:**
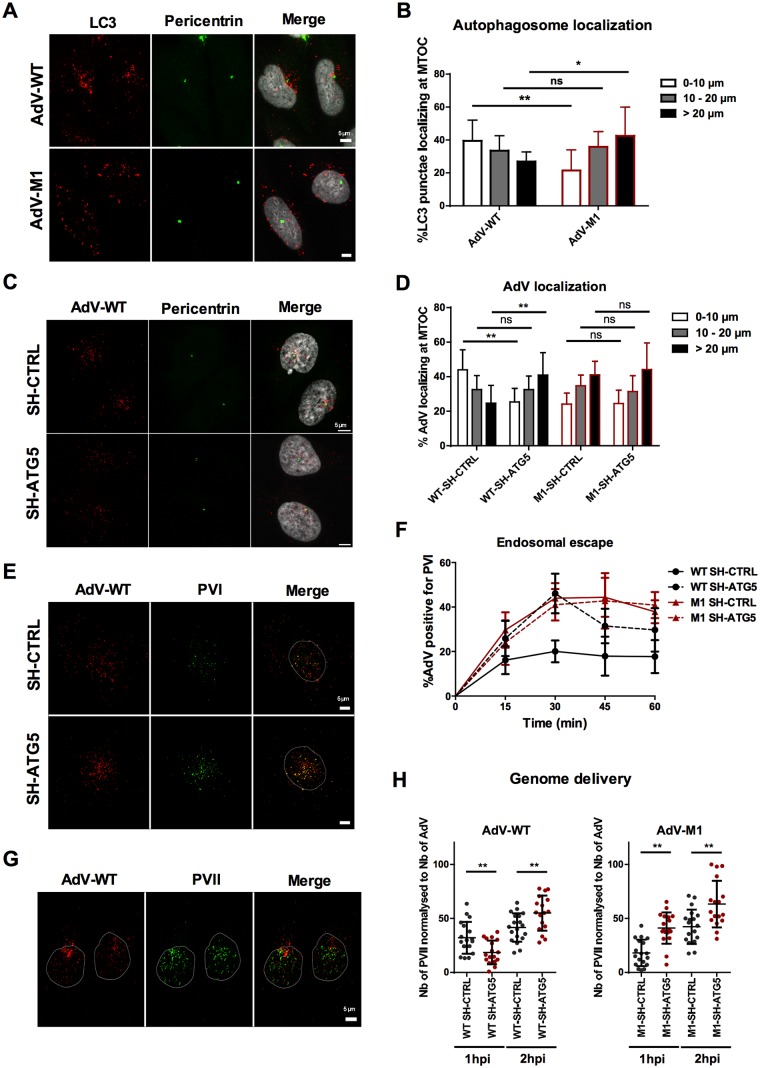
Nuclear transport of AdV involves the autophagic machinery. (A) Representative panel of WT or M1 infected cells at 1hpi stained with LC3 (red signal) and pericentrin (green signal) specific antibodies. (B) Quantification of the relative distribution of autophagosomes for experiment shown in (A) essentially analyzed as described for Fig 9E. (n>12 cells; NS: no significant; *: P<0.05; **: P<0.01) (C) Representative panel of WT infected cells at 1hpi depleted for ATG5 or control depleted (as indicated) and stained with AdV (red signal) and pericentrin (green signal) specific antibodies. (D) Quantification of the relative virus distribution as in (B) for the experiment shown in (C) including the distribution of M1 and WT viruses. (n>12 cells; NS: no significant, **: P<0.01) (E) Representative panel of WT infected cells at 1hpi depleted for ATG5 or control depleted (as indicated) and stained with AdV (red signal) and PVI (green signal) specific antibodies to mark PVI separation from the virus. (F) Infection time course analysis of PVI release from M1 (red line) vs. WT (black line) viruses in ATG5 depleted (dotted line) vs. control depleted cells (solid line). Shown is the percentage of PVI positive AdV at indicated time points. The errors bars are cell-to-cell variation (10 cells were analyzed for each conditions). (G) Representative panel of WT infected cells at 1hpi and stained with specific antibodies against AdV (red signal) and specific antibodies against PVII (green signal) to mark nuclear genomes. (H) Quantification of nuclear genome delivery. ATG5 and control depleted cells were infected with WT and M1 and fixed at 1 and 2hpi and stained for AdV and PVII. The number of nuclear PVII dots was calculated and normalized for virus particles at each condition as indicated below the graph (n>16 cells; **: P<0.01; ***: P<0.0001). (See also S7 Fig).

To address a possible role of autophagy in AdV nuclear transport we first infected cells with WT and M1 and analyzed the subcellular distribution of LC3 positive structures at 1hpi. This experiment should distinguish if MTOC accumulation of LC3 positive structures required recruitment of Nedd4.2 towards WT viruses or, in the case of the M1 virus, if availability of Nedd4.2 without capsid recruitment via protein VI was sufficient for MTOC targeting of LC3 (Fig 10A). WT infected cells showed increased MTOC accumulation of LC3 positive structures compared to M1 infected cells, which we confirmed by MTOC proximity quantification (Fig 10B). Because *in vivo* imaging of fluorescent viruses in LC3-GFP expressing cells often displayed virus mobility in association with LC3 (S7 Fig and S2 Movie) we asked if the autophagic machinery plays a more direct role in WT MTOC targeting. We repeated the above assay in ATG5 depleted cells and quantified the virus distribution around the MTOC. Most cells showed reduced MTOC accumulation for WT viruses in ATG5 depleted cells (Fig 10C), which we confirmed by MTOC proximity quantification. In contrast the more random distribution of M1 was not affected by ATG5 depletion (Fig 10D). Because the WT virus distribution in ATG5 depleted cells was very similar to the distribution of the M1 virus we asked if the endosomal escape was still functional. For this we compared PVI release between WT and M1 in ATG5 depleted cells vs. control cells as a measure of endosomal escape (Fig 10E). The analysis showed that ATG5 depletion did not impact on the initial release of PVI from either WT or M1 but delayed the separation of PVI from the WT (Fig 10F) showing that efficient endosomal escape requires ATG5.

Nuclear genome delivery can be considered as endpoint of AdV entry, which can be quantified by accumulation of genome associated protein VII dots in the nucleus [39] (Fig 10G). We quantified nuclear import of adenoviral genomes over time in ATG5 depleted and control cells for both viruses. Our analysis showed an initial delay for WT genomes in ATG5 depleted cells at 1 hpi that was fully compensated at 2 hpi confirming that fast nuclear genome transport relies on ATG5 while compensatory transport mechanisms exist (Fig 10G, left panel). It also confirmed that the M1 mutant virus was subject to autophagic degradation because ATG5 depletion increased nuclear genomes compared to control depleted cells suggesting that more virion particles survive the entry process and are able to deliver their genome. Nevertheless, nuclear genome arrival in ATG5 depleted cells showed the same delay as WT genomes (Fig 10G, right panel).

Taken together our study demonstrates how upon entry adenoviruses use a capsid encoded PPxY-peptide motif to exploit and control the autophagic machinery at multiple levels to secure efficient genome delivery.

## Discussion

Many invasive pathogens challenge the cellular membrane integrity to access the cytosol. Cells are able to sense pathogen invoked membrane damage via out-of-place detection of intra-lumenal glycans exposed to the cytosol. Thurston and co-workers showed that several galectins as well as poly-ubiquitin serve as danger sensor for ruptured membranes but only Gal8 via its specific adapter NDP52 restricts bacterial proliferation targeting the damaged vesicles for autophagic degradation [15]. Here we show that cells respond via a similar principle to incoming AdV demonstrating that non-enveloped endosomolytic viruses also activate selective autophagy through membrane damage. Under our assay conditions we show that release of the membrane lytic PVI from internalized viruses induced the transient association with galectins (3,8 and 9), ubiquitin, adapter molecules (p62 and NDP52) and LC3 within minutes of virus uptake. Using the control TS1 mutant virus, which lacks PVI release, we were able to pinpoint the membrane damage as the initiating event that causes this response. While WT viruses rapidly escaped from the ruptured endosome, and accumulate at the MTOC, the mutant M1 virus lacking a conserved PPxY motif in PVI remained associated with the ruptured (Gal3, Gal8 and PVI positive) vesicle and was subject to autophagic degradation. EM analysis revealed that during this process autophagosomal membranes engulf M1 viruses still associated with ruptured endosomes. Accordingly degradation through autophagy was able to explain the M1 infectivity defect and autophagy inhibition restored M1 infectivity. Thus we identified the capsid encoded PPxY motif as molecular determinant that allows the WT virus to subvert the cellular antiviral response providing the first example of a virus encoded motif to combat cellular antimicrobial autophagy. M1 virus removal occurred through a pathway involving Gal8 detection of virus induced membrane damage corroborating that autophagy recruitment through Gal8 is part of a conserved cellular pathway for the detection and removal of membrane damage evoked by microbes as previously suggested [15]. Gal3, which was also recruited to AdV ruptured membranes was not restricting the M1 mutant virus despite its recently described link to autophagy [40]. Depletion of the Gal8 binding autophagy receptor NDP52 as well as p62, which are both involved in restricting invasive bacteria was much less effective than autophagy inhibition or Gal8 depletion in protecting M1 from degradation. This observation suggests the existence of additional and/or alternative pathways to link AdV induced membrane damage via Gal8 towards autophagy clearance. These pathways may include the use of alternative autophagy receptors such as TAXBP1 recently shown to be involved in Salmonella clearance although no Gal8 binding was reported [41].

Our work with the M1 virus underpins the power of selective autophagy as antimicrobial mechanisms and shows how AdV have evolved capsid encoded evasive mechanism. This may not be true for all endosomolytic viruses. A recent report showed that picornavirus induced membrane damage also activates Gal8 mediated autophagy. In this case however the virus uses a cellular factor, the lipid-modifying enzyme PLA2G16, to counteract selective autophagy [42]. Remarkably, escaping endosomes would suffice for AdV to evade autophagic degradation. Nevertheless we observe that the WT virus uses the PPxY motif in PVI also to prevent efficient formation of autolysosomes. This effect is not linked to the onset of autophagy because LC3 punctae are induced and LC3-II conversion takes place upon WT infection. However, unlike with the M1 virus, we see much less large autophagosomal structures in WT infected cells and LC3-II levels rapidly return to basal levels. One possible explanation is that the WT virus interferes with the elongation process of the autophagosomal membrane to prevent autophagosome formation. This would make sense because only fully formed autophagosmes can fuse with lysosomes [43]. Depletion of Nedd4.2 removes the ability of the WT to interfere with autolysosome formation showing that the recruitment of Nedd4.2 through the PPxY motif in capsid protein PVI is central to AdV autophagy evasion. Murine Nedd4.2 was recently identified as factor that promotes autophagy using knockdown approaches [44]. In our hands Nedd4.2 depletion increases basal autophagy levels and also inhibits autolysosome formation independently of infection suggesting a key role for Nedd4.2 in physiological autophagy regulation. This observation is supported by recent work showing that Nedd4.2 is involved in controlling ULK1 levels under stress conditions to limit autophagy [45]. Diverting Nedd4.2 from its physiological role could therefore be a major function of the PVI PPxY motif. Another Nedd4 ligase, Nedd4.1, was recently shown to also regulate autophagy [46]. In addition, Nedd4.1 (but not Nedd4.2) was shown to control cellular Beclin-1 levels, a subunit of the class III phosphatidylinositol 3-kinase complex, which is crucial for the phagophore formation and membrane elongation [47,48]. We observe a stronger association of the M1 virus with PI3P positive membranes coinciding with enrichment of Beclin-1 on cellular membranes, suggesting that the WT selectively alters the function of the class III PI3K complex during entry although we were unable to functionally link this observation to Nedd4.2. Still, this seems to be a common viral strategy as several enveloped viruses target Beclin-1 and the class III PI3K complex to interfere with autophagic processes [49] including autophagosome maturation [50].

One attractive reason for viruses to control autolysosome formation is to limit antigenic presentation via the MHC-II pathway, which can be fed through autophagy [51]. E.g. Herpes simplex virus (HSV-1) employs the viral γ34.5 protein to bind and inactivate Beclin-1 to limit antigenic presentation [52]. AdV down regulates the antigen presenting machinery (MHC-I) During replication via regulatory E3 proteins [53,54]. Currently there is not a known mechanism underlying control of MHC-II presentation once transcription has begun although it might be an important obstacle. Our observations would suggest that using a capsid-encoded determinant, the PPxY motif, allows viral control of MHC-II antigen presentation upon entry preceding viral gene expression, which gives the virus an advantage over its host. In turn it identifies M1 capsids as potential tools for enhanced MHC-II exposure, which maybe of relevance for vaccination or other therapeutic approaches.

Another important observation in our study is the role of autophagy in virus transport towards the nucleus. We show that MTOC targeting for the WT requires dynein motors, Nedd4.2, the PVI PPxY motif and the LC3 conjugation system (ATG5). Removal of either component prevents MTOC accumulation, including a delay in genome delivery presumably because the WT becomes entrapped at the site of membrane rupture similar to the M1. WT and M1 virus do not differ in the kinetics with which they release PVI or acquire membrane damage sensors (Gal3, Gal8) nor does the turnover of membrane damage appear accelerated in either case. An attractive explanation for our observations would be that the LC3 conjugation system aids in motor recruitment towards the entrapped WT virus permitting efficient endosomal escape while Nedd4.2 recruitment via the PPxY motif in PVI interferes with the autophagosome maturation process initiated by the detection of damaged endosomes. A direct role for LC3 in microtubule transport during autophagosomal maturation and trafficking was recently established [21–23]. Thus PVI exposure could provide a higher affinity-binding site for Nedd4.2, which might redirect LC3 acquisition by molecular motor or motor scaffolding proteins for autophagic transport and maturation in favor of viral escape. Escape of the virus would not end the autophagosome maturation defect because (some) PVI remains within the ruptured membrane although the exact fate of the ruptured membrane including PVI after viral escape remains unclear at this stage.

**Fig 11:**
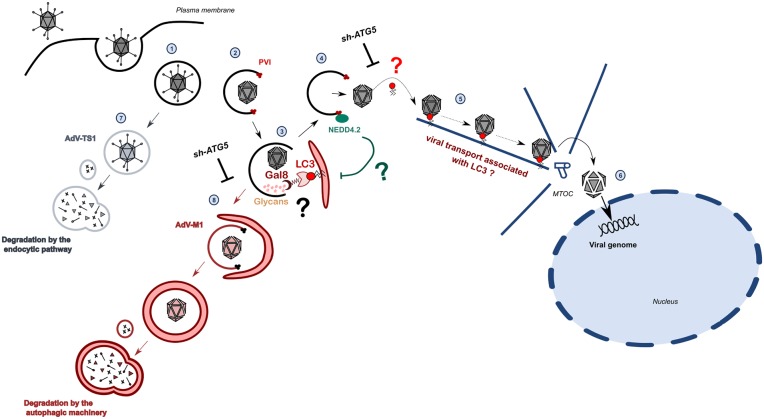
Model for adenovirus control of autophagic processes upon entry. AdV enter cells by receptor-mediated endocytosis (1) followed by partial disassembly to release the internal membrane lytic capsid protein PVI (2). PVI release initiates membrane rupture and intralumenal glycans are recognized via galectins and the autophagic machinery is recruited through Gal8 and LC3 to the damaged endosome mediated by yet to clarify adapter molecules (3). The recruitment of Nedd4.2 via the PPxY motif in PVI prevents formation of autophagosomes via an unknown mechanisms and facilitates endosomal escape (4). Endosomal escape involves the autophagic machinery because ATG5 depletion affects dissociation of virus from damaged vesicle. Dynein motor complexes are also required to access cytosolic microtubule mediated transport towards the MTOC which may occur in association with LC3 (5). Subsequent genome release occurs at the nuclear pore complex (6). Genome delivery is delayed upon ATG5 depletion. If PVI is not released (AdV-TS1), no membrane damage occurs and viruses are degraded via lysosomal sorting (7). If PVI is released and membrane damage occurs but the virus does not escape (AdV-M1), capsids are degraded via autophagy (8). This degradation is limited in absence of functional autophagy (e.g. upon ATG5 depletion).

Our preliminary assessment using *in vivo* imaging analysis suggest that WT virus may move from the membrane penetration site to the MTOC in association with LC3. Furthermore the depletion of LC3 conjugation impairs the MTOC accumulation of viral particles. It is thus possible that AdV acquire LC3 for its own transport to the nucleus. While recently autophagosomes where suggested to support AdV endosomal escape by fusing with endosomes [27] future work has to clarify if LC3 plays a more active role during endosomal escape e.g. as part of the motor complex, which extracts the virus from the endosome and/or as part of the complex that drives cytosolic transport of escaped viruses. Such a tentative model as detailed in Fig 11 would accommodate our observation that the WT on one hand limits autolysosome formation upon membrane damage while at the same time depletion of ATG5 prevents efficient endosomal escape and nuclear transport suggesting a reprogramming of autophagy to benefit the virus. It would also be in agreement with the observation that subsequent transport and genome delivery is delayed (but not abolished) in ATG5 depleted cells without affecting the overall accumulated infectivity measured at 24hpi. Delayed nuclear transport could also (in part) explain our previous observation how protein VI can affect transcription initiation of incoming viral genomes [55].

In summary our work has highlighted how a minimum of genetic conservation through preserving a short capsid encoded peptide motif allows incoming viruses (and presumably other pathogens) to subvert host cell defense mechanisms to profit at multiple layers. In this specific case the accumulated benefits for AdV include limited antigenic presentation paired with accelerated genome delivery showing that autophagy may act as a pro-viral mechanism upon entry of non-enveloped viruses. This is also the first direct demonstration how a viral pathogen overcomes the cellular response to membrane breach. Given the high prevalence of capsid encoded PPxY motifs in other viral systems (including HSV-1) [56–58] and a common association of viruses with the MTOC in entry it may be worthwhile to investigate if diverting Nedd4.2 during entry is part of a broader viral strategy for immune evasion and transport, which would make for an excellent drug target.

## Materials and methods

### Autophagy assays

All autophagy related assays were done in accordance to the “guidelines for the use and interpretation of assays for monitoring autophagy” (3^rd^ Edition) [34]

### Cell culture and virus production

All cells were grown under standard conditions (detailed in SI). ATG5 -/- and control MEFs were a kind gift from R. Duran, Institute Bergonié, Bordeaux, France. Amplification of human recombinant Ad5-VI-WT, Ad5-VI-M1 viruses and their E1-deleted GFP-transgene expressing vector counterparts (including Ad5-*ts1*-GFP) was done in HEK293 (Human Embryonic Kidney 293 cells, ATCC CRL-1573, kindly provided by G Nemerow, Scrips research institute, La Jolla, USA) cells and purified using double CsCl_2_-banding [10,11]. Virus particle to cell ratios were calculated based on the estimated copy numbers of viral genomes. Copy numbers were calculated according to the OD_260_ method (1 OD_260_ = 1.16×10^12^ particles/ml) [59]. Viruses were labeled by using the Alexa Fluor Microscale labeling kit (LifeTechnologies) as detailed in [26].

### Transfection, RNA interference and lentiviral transduction

Plasmid transfections was done in 12-well dishes using 1×10^5^ U2OS (Human bone Osteosarcoma Epithelial cells, ATCC HTB-96, kindly provided by M. Piechaczyk, IGMM, Montpellier, France) cells using Lipofectamine 2000 and OptiMEM (LifeTechnologies) according to the manufacturer’s instructions. RNAi mediated depletions were performed in 12-well dishes using 5×10^4^ U2OS cells. Following optimization cells were transfected after 24 and 48 h with 20 to 50 pmol of each siRNA constructs using Lipofectamine RNAimax and OptiMEM (LifeTechnologies). Depletion using shRNA-encoding lentiviruses was done by overnight incubation of 1×10^5^ U2OS cells (in 12-well dishes) with lentiviral particles at MOI of 5. The following day lentiviruses were removed and replaced by fresh complete DMEM and transduced cells were selected 24h later by adding puromycin (2μg/ml, Invitrogen). Sequences are listed in the SI.

### Adenoviral transduction and FACS analysis

Cell transduction with adenoviral vector was performed using 1×10^5^ U2OS cells seeded into 24-well plates. Cells were pre-treated with 100 μM CilioD for 30min or with 10mM of 3’MA or 50μM of Chloroquine for 3h or DMSO and DMF as vehicle control. Cells were transduced with 50 physical particles per cell of either GFP expressing WT or M1 in the presence of drugs followed by medium replacement with drug-free medium after 3h. Cells were analyzed 24h later by flow cytometry for GFP expression. Acquisitions were done on a FACSCantoII cytometer (BD Biosciences) and the data were processed and analyzed by the FACSDIVA software (BD Biosciences). U2OS depleted cells (siGAL3/8/9, siCTRL, shATG5/p62/NDP52 and shCTRL) were transduced following the same procedure.

### Western blot and antibodies

For western blot analysis cell lysates were separated on size-resolution adapted SDS-PAGE and transferred to nitrocellulose membranes (cut off of 0.2μm). Extraction of membrane proteins was done using the Mem-PER^™^ plus membrane protein extraction kit (ThermoFisher). Membranes were blocked in TBS containing 10% of dry-milk and 0.01% of Tween 20 (Sigma) during one hour at room temperature, followed by over-night incubation at 4°C with primary antibodies and with HRP-conjugated secondary antibodies against rabbit, goat or mouse (Sigma) at a dilution of 1∶10 000 for 1 one hour at room temperature. Specific signals were revealed using the enhanced chemiluminescence detection system (Super signal West femto, Thermoscientific) and signals were acquired using an ImageQuant LAS 4010 system (GE Healthcare life Sciences). Antibodies are listed in the SI.

### Time course of infection and immunofluorescence

For immunofluorescence analysis 1×10^5^ U2OS were grown on coverslips in a 12-well dish. Coverslips were incubated with 150μl of complete DMEM containing viruses (250 physical particles per cells) during 30 min at 37°C. Then viruses were removed and replaced by complete DMEM. Coverslips were fixed with 4% PFA at each time point (or in Methanol during 20 minutes at -20°C, for LC3 staining). Cells were blocked/permeabilized with IF-buffer (10% FCS in PBS and 0.5% Saponin). Primary antibody and secondary antibodies where applied to the coverslip in IF-buffer for 1 h at 37°C. Cells were mounted in DAKO mounting media containing DAPI and analyzed by confocal microscopy. Antibodies are listed in the SI.

### Confocal microscopy, live-cell imaging and image analysis

Confocal images were taken on a Leica SP5 confocal microscope equipped with Leica software and analyzed as detailed in SI. For quantitative image analysis we analyzed for each condition/time point at least 10 cells with >50 virus particles each (n>500). Live cell imaging was performed as described previously [39]. Briefly, cells were seeded in ibidi μ-slide VI^0.4^ (Ibidi), and images were acquired using a Leica spinning-disk microscopy system equipped with an environmental chamber heating the whole optical system to 37°C. Frames were taken every second (x100 objective) for each color channel and recorded using MetaMorph software and assembled in ImageJ.

### Transmission Electron Microscopy (TEM)

U2OS cells were grown to 70% confluency in 35mm glass bottom Grid-500 μ-Dish (Ibidi, Cat.No. 81168) and infected with Alexa594 fluorescently labeled WT or M1 viruses (250 physical particles per cell) at 37°C for 30 min. At 30 min post infection cells were fixed with 2.5% glutaraldehyde (GA) in PBS overnight at 4°C. Subsequently, the cells were washed with PBS, postfixed for 30 minutes with 1% OsO4 in PBS, washed with ddH2O, and stained with 1% uranyl acetate in water. The samples were gradually dehydrated with ethanol and embedded in Epon resin (Carl Roth, Germany) for sectioning. Ultrathin 50 nm sections were prepared using Ultracut Microtome (Leica Microsystems, Germany). The sections were poststained with 2% uranyl acetate. Electron micrographs were obtained with a 2K wide angle CCD camera (Veleta, Olympus Soft Imaging Solutions GmbH, Münster, Germany) attached to a FEI Tecnai G 20 Twin transmission electron microscope (FEI, Eindhoven, The Netherlands) at 80kv.

### ELIspot and ELISA assays

0.5-10x10^6^ monocyte-derived dendritic cells were derived from healthy anonymous donors and transduced with virus or media alone for 2 hours. Cells were incubated with CD4+ T-cell clones that had been cultured for 4 weeks to recognize a conserved AdV hexon epitope at a 3:1 ratio overnight. Supernatants were collected and ELISA was performed according to manufacturer’s instructions (eBioscience). For ELISpot assays, B6 mice (The Jackson Laboratory, Barr Harbor, ME) were infected with 10^10^ viral particles, or PBS control, intramuscularly. Mice were sacrificed 10 days after infection, and whole spenocytes were collected. Splenocytes were plated into ELIispot plates (Millipore) and stimulated with Ad5-luc capsids or GFP purified from *E*. *coli* for 48 hours. ELIspot was performed per manufacturer’s instructions (Millipore) and spots were reported as the number of spot forming colonies (SFC)/10^5 PBMCs.

Human monocyte-derived dendritic cells were cultured from peripheral blood mononuclear cells (PBMCs) obtained from healthy donors. Briefly, PBMCs were isolated from fresh whole blood by centrifugation on Histopaque cell separation medium. Monocytes were selected by adherence to culture dishes for 1 h, washed, and cultured in Iscove's modified Eagle's medium plus 10% FBS plus 25 ng/ml recombinant human GM-CSF (PeproTech) for 7 days to obtain differentiated dendritic cells. Human CD4+ T-cells recognizing a conserved epitope in adenovirus hexon were cloned as described previously [60]. Briefly, PBMC were suspended in 200 μl R10 media and incubated with 100 μM peptide for 1 h. Cells were diluted to 2 ml with R10 and incubated in 24-well plate at 37 C in a 5% CO2 atmosphere. After 7 days, human recombinant IL-2 (Becton Dickinson, Bedford, MA, USA) was added to a final concentration of 20 U/ml and then every 3–4 days. T-cell clones (1x10(6) per well) were restimulated with peptide-loaded, irradiated autologous PBMC (3000 rad) or (x10(6) per well) every 7–10 days. CD4+ T-cells were further isolated by negative selection magnetic cell sorting using a commercially available kit (Miltenyi Biotec, cat# 130-096-533).

### Ethics statement

All experiments using animals and human cells were conducted in accordance with the guidelines and under approval of the IACUC of Loyola University Chicago Stritch School of Medicine (#2011020) and the Loyola University Chicago Stritch School of Medicine Institutional Review Board (IRB # is LU204021) in accordance with guidelines set forth by the USDA and PHS Policy on Humane Care and Use of Laboratory Animals under the guidance of the Office of Laboratory Animal Welfare (OLAW). Loyola University Chicago, Health Sciences Division has an Animal Assurance on file with the Public Health Service (#A3117-01 approved through 02/28/2018), is a fully AAALAC International accredited institution (#000180, certification dated 11/19/2013), and is a USDA registered/licensed institution (#33-R-0024 through 08/24/2017). Loyola University Chicago, Health Sciences Division’s Institutional Animal Care and Use Committee (IACUC) is responsible for reviewing all protocols involving living vertebrate animals ensuring compliance with federal regulations, inspecting animal facilities and laboratories and overseeing training and educational programs.

### Statistical analysis

Data are presented as mean, error bars as standard deviation (STD) or standard error (SE) as indicated in the Fig. legend. Statistical analysis if not indicated otherwise was done using unpaired students t-test (NS: no significant, *:P<0.05; **:P<0.01; ***:P<0.001; ***: P<0.0001).

## Supporting information

S1 Fig Related to Fig 1: AdV endosomal escape is PPxY and dynein dependent. The human fetal lung fibroblast cell line MRC5, the human hepatocyte cells HepG2 and HuH7 and epithelial breast cancer cells MDA were infected with the indicated number of physical particles of either WT (black bars) or M1 (red bars) adenoviral vectors expressing GFP. Twenty-four hours post infection the percentage of GFP-positive cells was determined via FACS analysis and plotted for each cell line as indicated.
(TIFF)

S2 Fig Related to Fig 2. Recruitment of adaptor proteins upon entry. (A) Representative confocal images for the different viruses inducing membrane damage in cells marked by Gal8. U2OS cells were infected with viruses as indicated to the left of each panel, fixed at 30min post infection and stained with anti-AdV (red signal) and anti-Gal8 (green signal). (B) Comparative quantification of the absolute number of LC3 punctae per cell in Hela cells at 30 min post infection for the different viruses vs. control cells as indicated below each bar. (C) Representative confocal images essentially as in (A) using anti-AdV (red signal) and anti-NDP52 antibodies (green signal). (D) Representative confocal images essentially as in (A) using anti-AdV (red signal) and anti-p62 antibodies (green signal).
(TIFF)

S3 Fig Related to Fig 4: Increased association of AdV-M1 with PI3P platforms and autophagy adapter effect. (A) The left panel shows representative confocal images of U2OS cells transfected with a plasmid encoding *the in vivo* marker for Pi3P PX-GFP (green signal) and infected with WT or M1 viruses as indicated to the left of each row. One hour post infection cells were fixed and stained with AdV specific antibodies (red signal). The percentage of colocalization with PI3P platforms at 1hpi was quantified for each virus (right panel). The error bars show cell to cell variation (10 cells are analyzed per conditions,**: P<0.01). (B) U2OS cells were infected with WT or M1 viruses, fixed at 1 hour post infection and stained for AdV (red signal) and Beclin1 (green signal). (C) Membrane extract of infected U2OS cells were analyzed at 1 hour and 2 hours post infection by western blot using antibodies against Beclin1. Ponceau red staining of transferred proteins is shown as a loading control.
(TIFF)

S4 Fig Related to Fig 7: Efficient depletion of autophagy related factors. (A) Top panel: Stably Gal3-mCherry expressing cell were depleted with specific or control siRNA transfection as indicated above each lane. Depletion levels were detected with specific antibodies against mCherry and GAPDH as loading control as shown to the right. Bottom panel: U2OS cells were depleted with specific or control siRNAs as indicated above each lane and detected with Gal8 or Gal9 specific antibodies shown to the right. Tubulin specific antibodies were used as loading control. (B) Left panel: U2OS cells were depleted using lentiviral SH-RNA transduction as indicated above each lane followed by selection as detailed in the material and methods section. Depletion levels were detected by western blot with NDP52 or p62 specific antibodies as shown to the right. GAPDH specific antibodies were used as loading control. Right panel: U2OS cells were control- or NDP52- depleted as indicated using lentiviral SH-RNA transduction followed by si RNA transfection to deplete p62 where indicated. Depletion levels were detected with specific antibodies shown to the right.
(TIFF)

S5 Fig Related to Fig 8: AdV limits autophagy and prevents antigen presentation. (A) U2OS cells were pre-treated with 50μM chloroquine for 4 hours followed by infection with WT or M1 viruses. Cell lysates were harvested at indicated time points and analyzed by western blot using LC3 and GAPDH specific antibodies as shown to the left. (NI = non infected). The ratio of LC3II/GAPDH normalized to the non-infected condition was determined and is given below the panel. (B) Representative panel of confocal images from cells transduced with optimized amounts of lentivirus encoding tandem GFP-RFP-LC3 and either treated with chloroquine (50μM for 4hours) or infected for 1h with WT or M1 viruses as indicated. (C) The ratio between autophagosomes (double positive punctae, GFP+ and RFP+) and autolysosomes (single positive punctae, GFP- and RFP+) for the experiment shown in (B) was calculated for WT and M1 infected cells as indicated (n>15 cells; **: P<0.01.). (D) Human monocyte derived dendritic cells were transduced with WT or M1 for 18 hours. Cell surface expression of HLA-DR or CD86 was assessed by FACS and is shown for infected and control cells as indicated.
(TIFF)

S6 Fig Related to Fig 9: Nedd4.2 controls autophagosome maturation upon starvation. (A) Nedd4.2 expression levels were determined by western blot analysis in Nedd4.2 and control depleted cells using specific antibodies against Nedd4.2 and tubulin as loading control. (B) Representative panel of confocal images from Nedd4.2 or control depleted cells following overnight starvation in HBSS (indicated to the left) and stained with Lamp2 (magenta signal) and LC3 (green signal) specific antibodies. The detail corresponds to the boxed region. Note that autolysosomes appear white. (C) Quantification of LC3 punctae in starved vs. non-starved control cells either Nedd4.2 or control depleted (as indicated below the graph). (D) Experiment as in (C) showing the percentage of LC3 punctae also positive for Lamp2. (n>15 cells; NS: no significant; *: P<0.05; **: P<0.01; ****: P<0.0001)
(TIFF)

S7 Fig Related to Fig 10: Intracellular trafficking of LC3-positive AdV. (**A**) Live-cell imaging showing intracellular trafficking of LC3 positive WT virus to the perinuclear region. Stable expressing U2OS-LC3-GFP cells were infected with Alexa594 coupled WT viruses and imaged using spinning-disk confocal microscopy. The images show individual frames separated by ~45 seconds from Supplemental S2 Movie. The arrow points to a virus that is LC3 positive (left panel) and moves towards the perinuclear region (middle and right panel).
(TIFF)

S1 Movie Related to Fig 4: AdV acquires LC3 in living cells. Live-cell imaging showing how fluorescent WT virus acquires an LC3 coat close to the cellular periphery. Stable expressing U2OS-LC3-GFP cells were infected with Alexa594 coupled WT viruses and imaged using spinning-disk confocal microscopy at a rate of 1 frame/sec for each channel. Raw data were recorded in MetaMorph and assembled and processed using ImageJ software.
(AVI)

S2 Movie Related to Fig 10: Intracellular trafficking of LC3-positive AdV. Live-cell imaging showing intracellular trafficking of LC3 positive WT virus to the perinuclear region. Stable expressing U2OS-LC3-GFP cells were infected with Alexa594 coupled WT viruses and imaged using spinning-disk confocal microscopy at a rate of 1 frame/sec for each channel. Raw data were recorded in MetaMorph and assembled and processed using ImageJ software.
(AVI)

S1 File The supplemental information contains supplemental experimental procedures. (DOCX)

## References

[1] Koyama S, Ishii KJ, Coban C, Akira S. Innate immune response to viral infection. Cytokine. 2008 9;43(3):336–41. 10.1016/j.cyto.2008.07.009 18694646

[2] Nemerow GR, Stewart PL. Role of alpha(v) integrins in adenovirus cell entry and gene delivery. Microbiol Mol Biol Rev MMBR. 1999 9;63(3):725–34. 10477314 10.1128/mmbr.63.3.725-734.1999 PMC103752

[3] Wickham TJ, Mathias P, Cheresh DA, Nemerow GR. Integrins αvβ3 and αvβ5 promote adenovirus internalization but not virus attachment. Cell. 1993 4 23;73(2):309–19. 8477447 10.1016/0092-8674(93)90231-e

[4] Greber UF, Willetts M, Webster P, Helenius A. Stepwise dismantling of adenovirus 2 during entry into cells. Cell. 1993 11 5;75(3):477–86. 8221887 10.1016/0092-8674(93)90382-z

[5] Maier O, Marvin SA, Wodrich H, Campbell EM, Wiethoff CM. Spatiotemporal dynamics of adenovirus membrane rupture and endosomal escape. J Virol. 2012 10;86(19):10821–8. 10.1128/JVI.01428-12 22855481 PMC3457294

[6] Wiethoff CM, Wodrich H, Gerace L, Nemerow GR. Adenovirus protein VI mediates membrane disruption following capsid disassembly. J Virol. 2005 2;79(4):1992–2000. 10.1128/JVI.79.4.1992-2000.2005 15681401 PMC546575

[7] Leopold PL, Crystal RG. Intracellular trafficking of adenovirus: Many means to many ends. Adv Drug Deliv Rev. 2007 8 10;59(8):810–21. 10.1016/j.addr.2007.06.007 17707546

[8] Strunze S, Engelke MF, Wang I-H, Puntener D, Boucke K, Schleich S, et al Kinesin-1-mediated capsid disassembly and disruption of the nuclear pore complex promote virus infection. Cell Host Microbe. 2011 9 15;10(3):210–23. 10.1016/j.chom.2011.08.010 21925109

[9] Gastaldelli M, Imelli N, Boucke K, Amstutz B, Meier O, Greber UF. Infectious adenovirus type 2 transport through early but not late endosomes. Traffic Cph Den. 2008 12;9(12):2265–78. 10.1111/j.1600-0854.2008.00835.x 18980614

[10] Martinez R, Schellenberger P, Vasishtan D, Aknin C, Austin S, Dacheux D, et al The amphipathic helix of adenovirus capsid protein VI contributes to penton release and postentry sorting. J Virol. 2015 2;89(4):2121–35. 10.1128/JVI.02257-14 25473051 PMC4338868

[11] Wodrich H, Henaff D, Jammart B, Segura-Morales C, Seelmeir S, Coux O, et al A capsid-encoded PPxY-motif facilitates adenovirus entry. PLoS Pathog. 2010 3;6(3):e1000808 10.1371/journal.ppat.1000808 20333243 PMC2841620

[12] Austin S, Taouji S, Chevet E, Wodrich H, Rayne F. Using AlphaScreen to Identify Small-Molecule Inhibitors Targeting a Conserved Host-Pathogen Interaction. Methods Mol Biol Clifton NJ. 2016;1449:453–67. 10.1007/978-1-4939-3756-1_30 27613056

[13] Nabi IR, Shankar J, Dennis JW. The galectin lattice at a glance. J Cell Sci. 2015 7 1;128(13):2213–9. 10.1242/jcs.151159 26092931

[14] Paz I, Sachse M, Dupont N, Mounier J, Cederfur C, Enninga J, et al Galectin-3, a marker for vacuole lysis by invasive pathogens. Cell Microbiol. 2010 4 1;12(4):530–44. 10.1111/j.1462-5822.2009.01415.x 19951367

[15] Thurston TLM, Wandel MP, von Muhlinen N, Foeglein A, Randow F. Galectin 8 targets damaged vesicles for autophagy to defend cells against bacterial invasion. Nature. 2012 2 16;482(7385):414–8. 10.1038/nature10744 22246324 PMC3343631

[16] Dupont N, Lacas-Gervais S, Bertout J, Paz I, Freche B, Van Nhieu GT, et al Shigella phagocytic vacuolar membrane remnants participate in the cellular response to pathogen invasion and are regulated by autophagy. Cell Host Microbe. 2009 8 20;6(2):137–49. 10.1016/j.chom.2009.07.005 19683680

[17] von Muhlinen N, Akutsu M, Ravenhill BJ, Foeglein Á, Bloor S, Rutherford TJ, et al LC3C, bound selectively by a noncanonical LIR motif in NDP52, is required for antibacterial autophagy. Mol Cell. 2012 11 9;48(3):329–42. 10.1016/j.molcel.2012.08.024 23022382 PMC3510444

[18] Mizushima N, Levine B, Cuervo AM, Klionsky DJ. Autophagy fights disease through cellular self-digestion. Nature. 2008 2 28;451(7182):1069–75. 10.1038/nature06639 18305538 PMC2670399

[19] Itakura E, Mizushima N. Characterization of autophagosome formation site by a hierarchical analysis of mammalian Atg proteins. Autophagy. 2010 8;6(6):764–76. 10.4161/auto.6.6.12709 20639694 PMC3321844

[20] Nakatogawa H. Two ubiquitin-like conjugation systems that mediate membrane formation during autophagy. Essays Biochem. 2013;55:39–50. 10.1042/bse0550039 24070470

[21] Fu M, Nirschl JJ, Holzbaur ELF. LC3 Binding to the Scaffolding Protein JIP1 Regulates Processive Dynein-Driven Transport of Autophagosomes. Dev Cell. 2014 6 9;29(5):577–90. 10.1016/j.devcel.2014.04.015 24914561 PMC4109720

[22] Kimura S, Noda T, Yoshimori T. Dynein-dependent movement of autophagosomes mediates efficient encounters with lysosomes. Cell Struct Funct. 2008;33(1):109–22. 18388399 10.1247/csf.08005

[23] Pankiv S, Johansen T. FYCO1: linking autophagosomes to microtubule plus end-directing molecular motors. Autophagy. 2010 5;6(4):550–2. 10.4161/auto.6.4.11670 20364109

[24] Gomes LC, Dikic I. Autophagy in antimicrobial immunity. Mol Cell. 2014 4 24;54(2):224–33. 10.1016/j.molcel.2014.03.009 24766886

[25] Boyle KB, Randow F. The role of “eat-me” signals and autophagy cargo receptors in innate immunity. Curr Opin Microbiol. 2013 6;16(3):339–48. 10.1016/j.mib.2013.03.010 23623150

[26] Martinez R, Burrage AM, Wiethoff CM, Wodrich H. High temporal resolution imaging reveals endosomal membrane penetration and escape of adenoviruses in real time. Methods Mol Biol Clifton NJ. 2013;1064:211–26. 10.1007/978-1-62703-601-6_15 PMC4199227 23996260

[27] Zeng X, Carlin CR. Host Cell Autophagy Modulates Early Stages of Adenovirus Infections in Airway Epithelial Cells. J Virol. 2013 2;87(4):2307–19. 10.1128/JVI.02014-12 23236070 PMC3571477

[28] Klein SR, Jiang H, Hossain MB, Fan X, Gumin J, Dong A, et al Critical Role of Autophagy in the Processing of Adenovirus Capsid-Incorporated Cancer-Specific Antigens. PloS One. 2016;11(4):e0153814 10.1371/journal.pone.0153814 27093696 PMC4836716

[29] Paul P, Münz C. Autophagy and Mammalian Viruses: Roles in Immune Response, Viral Replication, and Beyond. Adv Virus Res. 2016;95:149–95. 10.1016/bs.aivir.2016.02.002 27112282

[30] English L, Chemali M, Duron J, Rondeau C, Laplante A, Gingras D, et al Autophagy enhances the presentation of endogenous viral antigens on MHC class I molecules during HSV-1 infection. Nat Immunol. 2009 5;10(5):480–7. 10.1038/ni.1720 19305394 PMC3885169

[31] Paludan C, Schmid D, Landthaler M, Vockerodt M, Kube D, Tuschl T, et al Endogenous MHC class II processing of a viral nuclear antigen after autophagy. Science. 2005 1 28;307(5709):593–6. 10.1126/science.1104904 15591165

[32] Lennemann NJ, Coyne CB. Catch me if you can: the link between autophagy and viruses. PLoS Pathog. 2015 3;11(3):e1004685 10.1371/journal.ppat.1004685 25811485 PMC4374752

[33] Bremner KH, Scherer J, Yi J, Vershinin M, Gross SP, Vallee RB. Adenovirus transport via direct interaction of cytoplasmic dynein with the viral capsid hexon subunit. Cell Host Microbe. 2009 12 17;6(6):523–35. 10.1016/j.chom.2009.11.006 20006841 PMC2810746

[34] Strunze S, Trotman LC, Boucke K, Greber UF. Nuclear Targeting of Adenovirus Type 2 Requires CRM1-mediated Nuclear Export. Mol Biol Cell. 2005 6;16(6):2999–3009. 10.1091/mbc.E05-02-0121 15814838 PMC1142442

[35] Klionsky DJ, Abdalla FC, Abeliovich H, Abraham RT, Acevedo-Arozena A, Adeli K, et al Guidelines for the use and interpretation of assays for monitoring autophagy. Autophagy. 2012 4;8(4):445–544. 10.4161/auto.19496 22966490 PMC3404883

[36] Bosse JB, Ragues J, Wodrich H. Fast generation of stable cell lines expressing fluorescent marker molecules to study pathogen induced processes. Methods Mol Biol Clifton NJ. 2013;1064:153–69. 10.1007/978-1-62703-601-6_11 23996256

[37] Roberts R, Ktistakis NT. Omegasomes: PI3P platforms that manufacture autophagosomes. Essays Biochem. 2013;55:17–27. 10.1042/bse0550017 24070468

[38] Brandenburg B, Zhuang X. Virus trafficking—learning from single-virus tracking. Nat Rev Microbiol. 2007 3;5(3):197–208. 10.1038/nrmicro1615 17304249 PMC2740720

[39] Komatsu T, Dacheux D, Kreppel F, Nagata K, Wodrich H. A Method for Visualization of Incoming Adenovirus Chromatin Complexes in Fixed and Living Cells. PloS One. 2015;10(9):e0137102 10.1371/journal.pone.0137102 26332038 PMC4557953

[40] Chauhan S, Kumar S, Jain A, Ponpuak M, Mudd MH, Kimura T, et al TRIMs and Galectins Globally Cooperate and TRIM16 and Galectin-3 Co-direct Autophagy in Endomembrane Damage Homeostasis. Dev Cell. 2016 10 10;39(1):13–27. 10.1016/j.devcel.2016.08.003 27693506 PMC5104201

[41] Tumbarello DA, Manna PT, Allen M, Bycroft M, Arden SD, Kendrick-Jones J, et al The Autophagy Receptor TAX1BP1 and the Molecular Motor Myosin VI Are Required for Clearance of Salmonella Typhimurium by Autophagy. PLoS Pathog [Internet]. 2015 10 9 [cited 2016 Aug 3];11(10). Available from: http://www.ncbi.nlm.nih.gov/pmc/articles/PMC4599966/10.1371/journal.ppat.1005174 PMC4599966 26451915

[42] Staring J, von Castelmur E, Blomen VA, van den Hengel LG, Brockmann M, Baggen J, et al PLA2G16 represents a switch between entry and clearance of Picornaviridae. Nature. 2017 1 19;541(7637):412–6. 10.1038/nature21032 28077878

[43] Ganley IG. Autophagosome maturation and lysosomal fusion. Essays Biochem. 2013;55:65–78. 10.1042/bse0550065 24070472

[44] Wang H, Sun R-Q, Camera D, Zeng X-Y, Jo E, Chan SMH, et al Endoplasmic reticulum stress up-regulates Nedd4-2 to induce autophagy. FASEB J Off Publ Fed Am Soc Exp Biol. 2016 7;30(7):2549–56. 10.1096/fj.201500119 27022162

[45] Nazio F, Carinci M, Valacca C, Bielli P, Strappazzon F, Antonioli M, et al Fine-tuning of ULK1 mRNA and protein levels is required for autophagy oscillation. J Cell Biol. 2016 12 19;215(6):841–56. 10.1083/jcb.201605089 27932573 PMC5166502

[46] Sun A, Wei J, Childress C, Shaw JH Iv, Peng K, Shao G, et al The E3 ubiquitin ligase NEDD4 is an LC3-interactive protein and regulates autophagy. Autophagy. 2017 1 13;1–16. 10.1080/15548627.2016.1268301 PMC5361608 28085563

[47] Platta HW, Abrahamsen H, Thoresen SB, Stenmark H. Nedd4-dependent lysine-11-linked polyubiquitination of the tumour suppressor Beclin 1. Biochem J. 2012 1 1;441(1):399–406. 10.1042/BJ20111424 21936852 PMC3242507

[48] Sun T, Li X, Zhang P, Chen W-D, Zhang H, Li D-D, et al Acetylation of Beclin 1 inhibits autophagosome maturation and promotes tumour growth. Nat Commun. 2015;6:7215 10.1038/ncomms8215 26008601 PMC4455096

[49] Münz C. Beclin-1 targeting for viral immune escape. Viruses. 2011 7;3(7):1166–78. 10.3390/v3071166 21994775 PMC3185790

[50] Liang Q, Chang B, Brulois KF, Castro K, Min C-K, Rodgers MA, et al Kaposi’s sarcoma-associated herpesvirus K7 modulates Rubicon-mediated inhibition of autophagosome maturation. J Virol. 2013 11;87(22):12499–503. 10.1128/JVI.01898-13 24027317 PMC3807930

[51] Münz C. Antigen Processing for MHC Class II Presentation via Autophagy. Front Immunol. 2012;3:9 10.3389/fimmu.2012.00009 22566895 PMC3342365

[52] Gobeil PAM, Leib DA. Herpes simplex virus γ34.5 interferes with autophagosome maturation and antigen presentation in dendritic cells. mBio. 2012;3(5):e00267–212. 10.1128/mBio.00267-12 23073763 PMC3470650

[53] Burgert HG, Maryanski JL, Kvist S. “E3/19K” protein of adenovirus type 2 inhibits lysis of cytolytic T lymphocytes by blocking cell-surface expression of histocompatibility class I antigens. Proc Natl Acad Sci U S A. 1987 3;84(5):1356–60. 2950523 10.1073/pnas.84.5.1356 PMC304428

[54] Windheim M, Hilgendorf A, Burgert HG. Immune evasion by adenovirus E3 proteins: exploitation of intracellular trafficking pathways. Curr Top Microbiol Immunol. 2004;273:29–85. 14674598 10.1007/978-3-662-05599-1_2

[55] Schreiner S, Martinez R, Groitl P, Rayne F, Vaillant R, Wimmer P, et al Transcriptional activation of the adenoviral genome is mediated by capsid protein VI. PLoS Pathog. 2012 2;8(2):e1002549 10.1371/journal.ppat.1002549 22427750 PMC3303589

[56] Garcia ML, Reynolds TD, Mothes W, Robek MD. Functional characterization of the putative hepatitis B virus core protein late domain using retrovirus chimeras. PloS One. 2013;8(8):e72845 10.1371/journal.pone.0072845 24009707 PMC3756966

[57] Irie T, Licata JM, Harty RN. Functional characterization of Ebola virus L-domains using VSV recombinants. Virology. 2005 6 5;336(2):291–8. 10.1016/j.virol.2005.03.027 15892969 PMC2929245

[58] Ushijima Y, Koshizuka T, Goshima F, Kimura H, Nishiyama Y. Herpes Simplex Virus Type 2 UL56 Interacts with the Ubiquitin Ligase Nedd4 and Increases Its Ubiquitination. J Virol. 2008 6;82(11):5220–33. 10.1128/JVI.02515-07 18353951 PMC2395212

[59] Segura-Morales C, Pescia C, Chatellard-Causse C, Sadoul R, Bertrand E, Basyuk E. Tsg101 and Alix interact with murine leukemia virus Gag and cooperate with Nedd4 ubiquitin ligases during budding. J Biol Chem. 2005 7 22;280(29):27004–12. 10.1074/jbc.M413735200 15908698

[60] Tang J, Olive M, Champagne K, Flomenberg N, Eisenlohr L, Hsu S, et al Adenovirus hexon T-cell epitope is recognized by most adults and is restricted by HLA DP4, the most common class II allele. Gene Ther. 2004 9;11(18):1408–15. 10.1038/sj.gt.3302316 15269714

