# Supplementary figures and images for: Multi-layered control of Galectin-8 mediated autophagy during adenovirus cell entry through a conserved PPxY motif in the viral capsid
Source: PLoS Pathog. 2017 Feb 13;13(2):e1006217. doi: 10.1371/journal.ppat.1006217 (PMC5325606; doi:10.1371/journal.ppat.1006217)

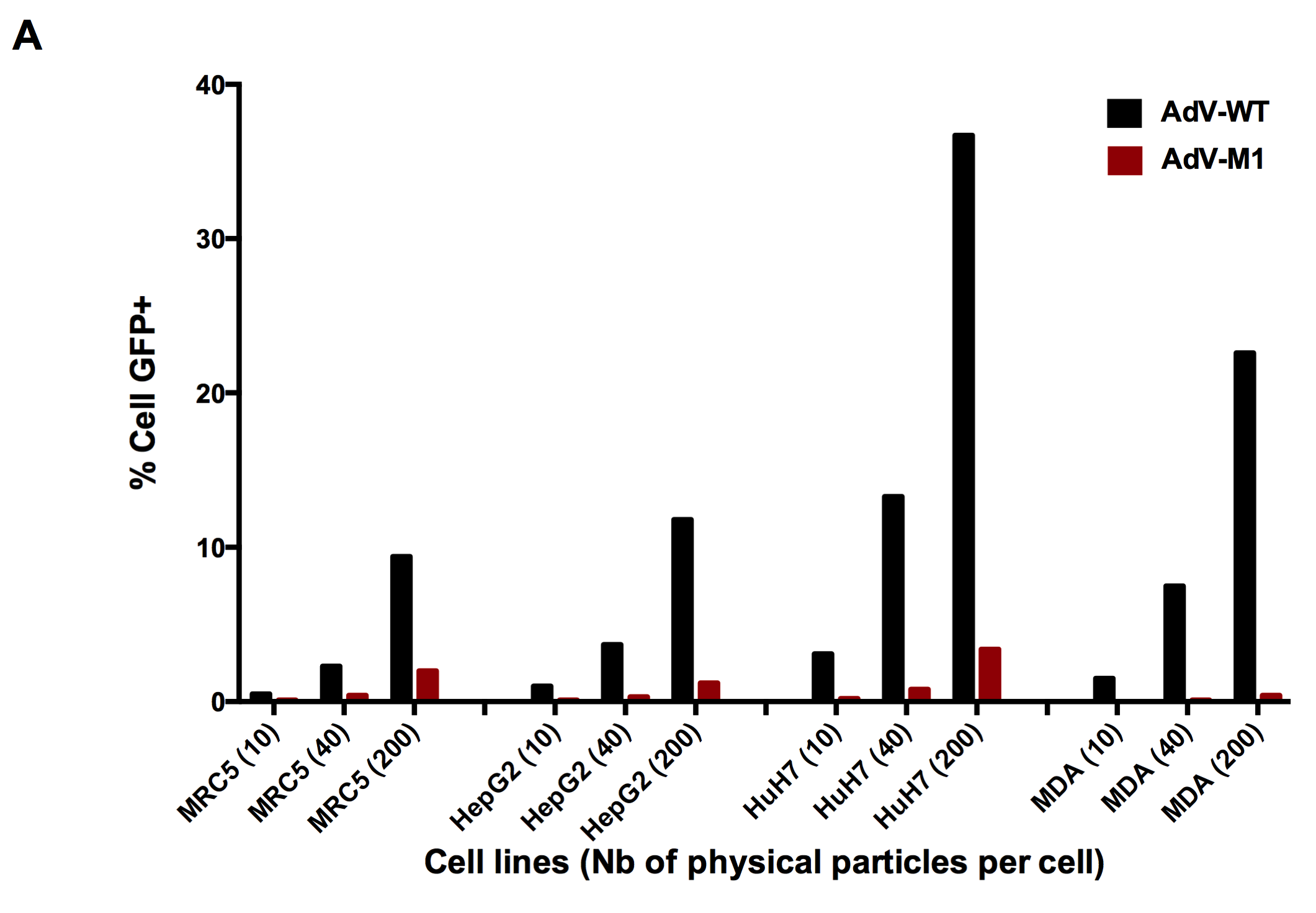

Supplement: S1 Fig — The human fetal lung fibroblast cell line MRC5, the human hepatocyte cells HepG2 and HuH7 and epithelial breast cancer cells MDA were infected with the indicated number of physical particles of either WT (black bars) or M1 (red bars) adenoviral vectors expressing GFP. Twenty-four hours post infection the percentage of GFP-positive cells was determined via FACS analysis and plotted for each cell line as indicated. (TIFF) [file ppat.1006217.s001.tiff]

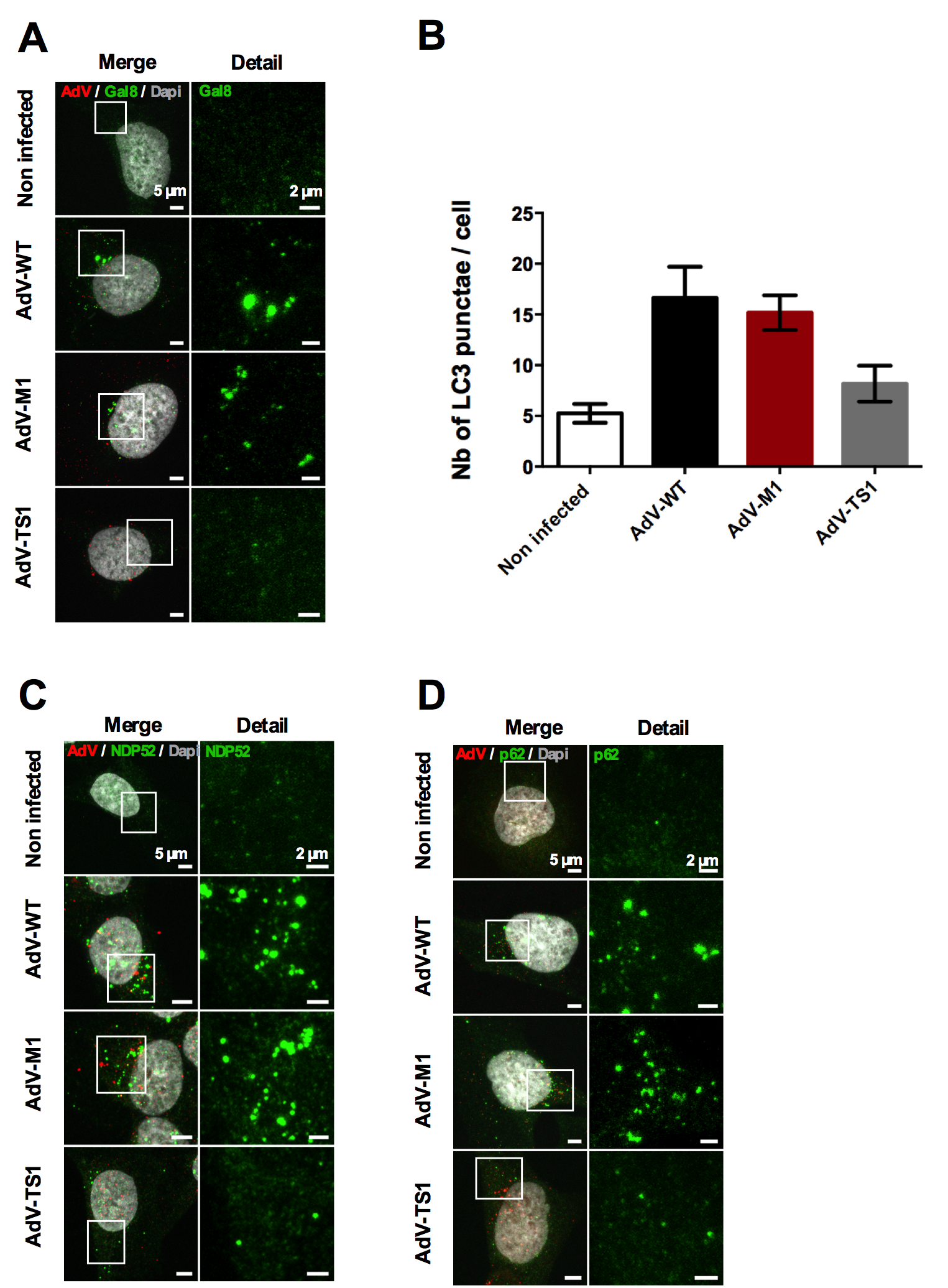

Supplement: S2 Fig — (A) Representative confocal images for the different viruses inducing membrane damage in cells marked by Gal8. U2OS cells were infected with viruses as indicated to the left of each panel, fixed at 30min post infection and stained with anti-AdV (red signal) and anti-Gal8 (green signal). (B) Comparative quantification of the absolute number of LC3 punctae per cell in Hela cells at 30 min post infection for the different viruses vs. control cells as indicated below each bar. (C) Representative confocal images essentially as in (A) using anti-AdV (red signal) and anti-NDP52 antibodies (green signal). (D) Representative confocal images essentially as in (A) using anti-AdV (red signal) and anti-p62 antibodies (green signal). (TIFF) [file ppat.1006217.s002.tiff]

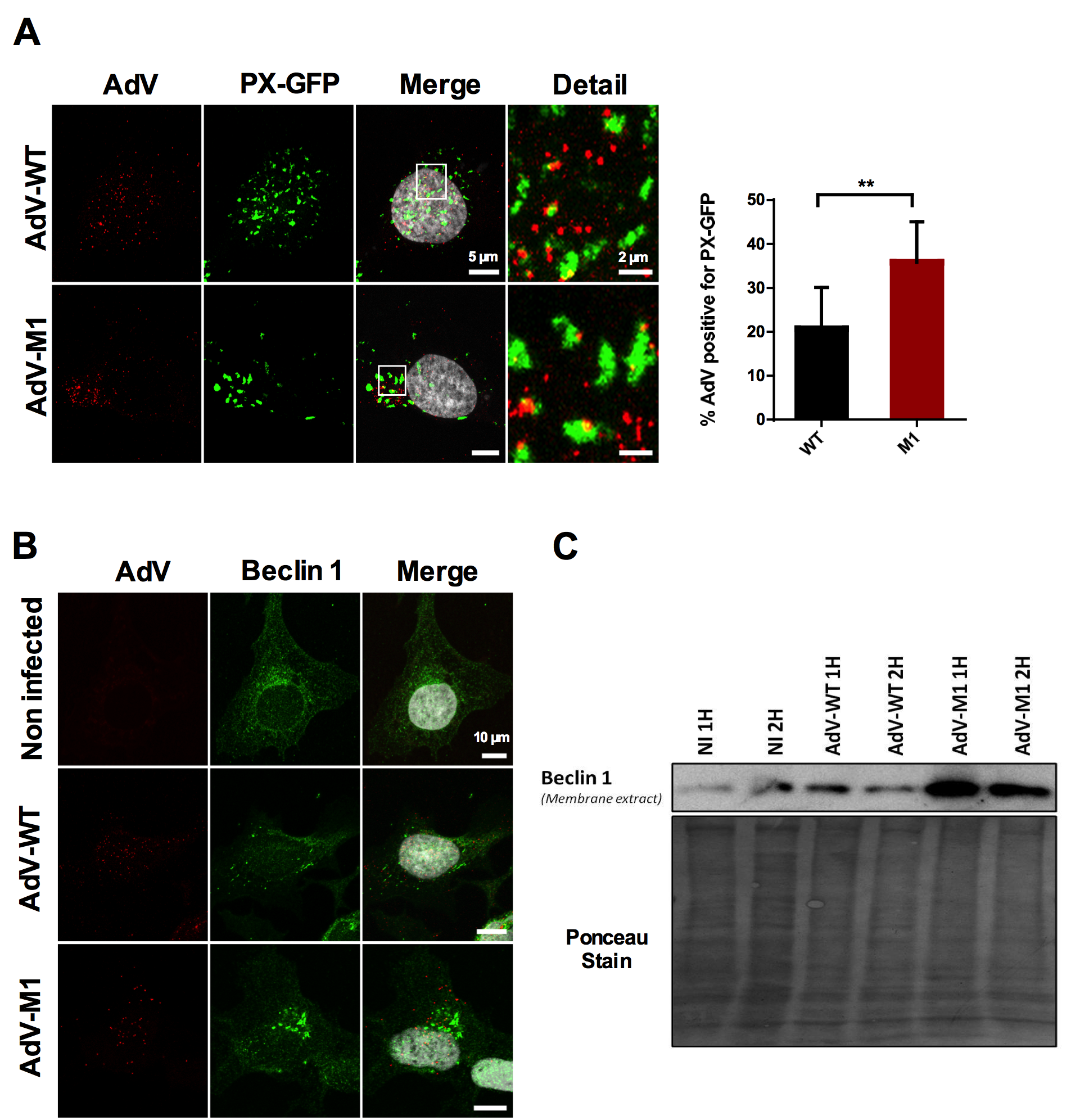

Supplement: S3 Fig — (A) The left panel shows representative confocal images of U2OS cells transfected with a plasmid encoding the in vivo marker for Pi3P PX-GFP (green signal) and infected with WT or M1 viruses as indicated to the left of each row. One hour post infection cells were fixed and stained with AdV specific antibodies (red signal). The percentage of colocalization with PI3P platforms at 1hpi was quantified for each virus (right panel). The error bars show cell to cell variation (10 cells are analyzed per conditions,**: P<0.01). (B) U2OS cells were infected with WT or M1 viruses, fixed at 1 hour post infection and stained for AdV (red signal) and Beclin1 (green signal). (C) Membrane extract of infected U2OS cells were analyzed at 1 hour and 2 hours post infection by western blot using antibodies against Beclin1. Ponceau red staining of transferred proteins is shown as a loading control. (TIFF) [file ppat.1006217.s003.tiff]

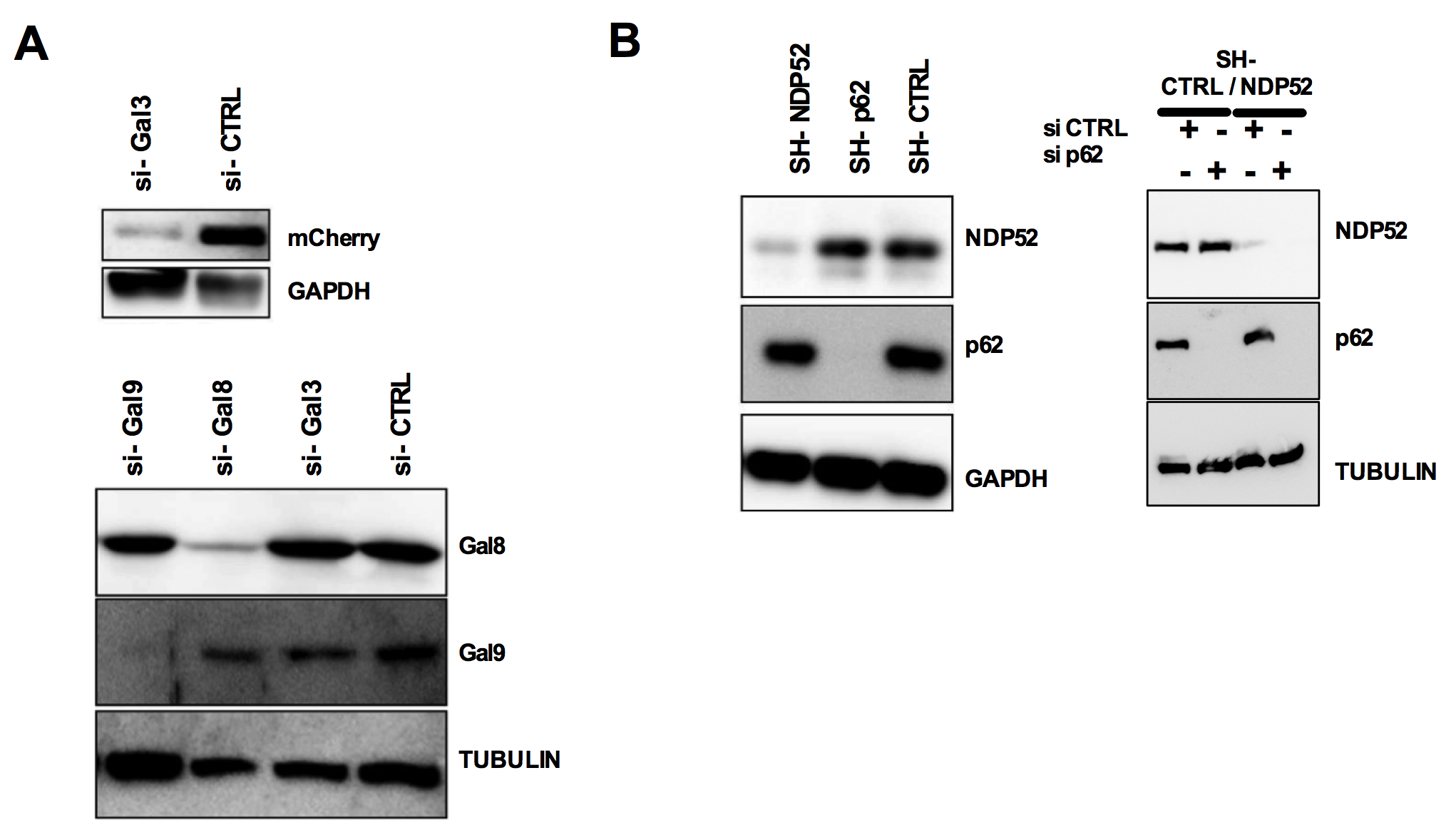

Supplement: S4 Fig — (A) Top panel: Stably Gal3-mCherry expressing cell were depleted with specific or control siRNA transfection as indicated above each lane. Depletion levels were detected with specific antibodies against mCherry and GAPDH as loading control as shown to the right. Bottom panel: U2OS cells were depleted with specific or control siRNAs as indicated above each lane and detected with Gal8 or Gal9 specific antibodies shown to the right. Tubulin specific antibodies were used as loading control. (B) Left panel: U2OS cells were depleted using lentiviral SH-RNA transduction as indicated above each lane followed by selection as detailed in the material and methods section. Depletion levels were detected by western blot with NDP52 or p62 specific antibodies as shown to the right. GAPDH specific antibodies were used as loading control. Right panel: U2OS cells were control- or NDP52- depleted as indicated using lentiviral SH-RNA transduction followed by si RNA transfection to deplete p62 where indicated. Depletion levels were detected with specific antibodies shown to the right. (TIFF) [file ppat.1006217.s004.tiff]

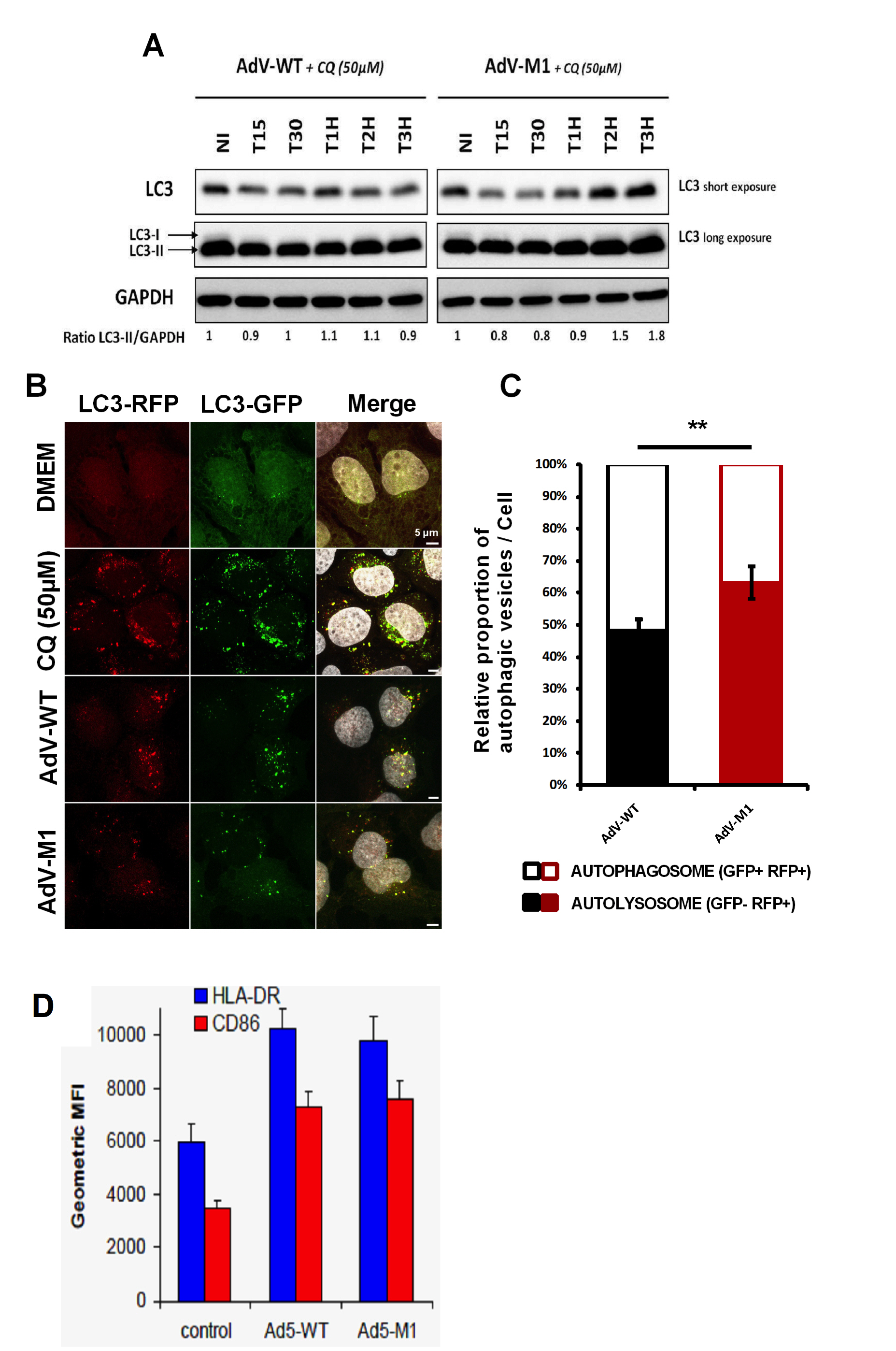

Supplement: S5 Fig — (A) U2OS cells were pre-treated with 50μM chloroquine for 4 hours followed by infection with WT or M1 viruses. Cell lysates were harvested at indicated time points and analyzed by western blot using LC3 and GAPDH specific antibodies as shown to the left. (NI = non infected). The ratio of LC3II/GAPDH normalized to the non-infected condition was determined and is given below the panel. (B) Representative panel of confocal images from cells transduced with optimized amounts of lentivirus encoding tandem GFP-RFP-LC3 and either treated with chloroquine (50μM for 4hours) or infected for 1h with WT or M1 viruses as indicated. (C) The ratio between autophagosomes (double positive punctae, GFP+ and RFP+) and autolysosomes (single positive punctae, GFP- and RFP+) for the experiment shown in (B) was calculated for WT and M1 infected cells as indicated (n>15 cells; **: P<0.01.). (D) Human monocyte derived dendritic cells were transduced with WT or M1 for 18 hours. Cell surface expression of HLA-DR or CD86 was assessed by FACS and is shown for infected and control cells as indicated. (TIFF) [file ppat.1006217.s005.tiff]

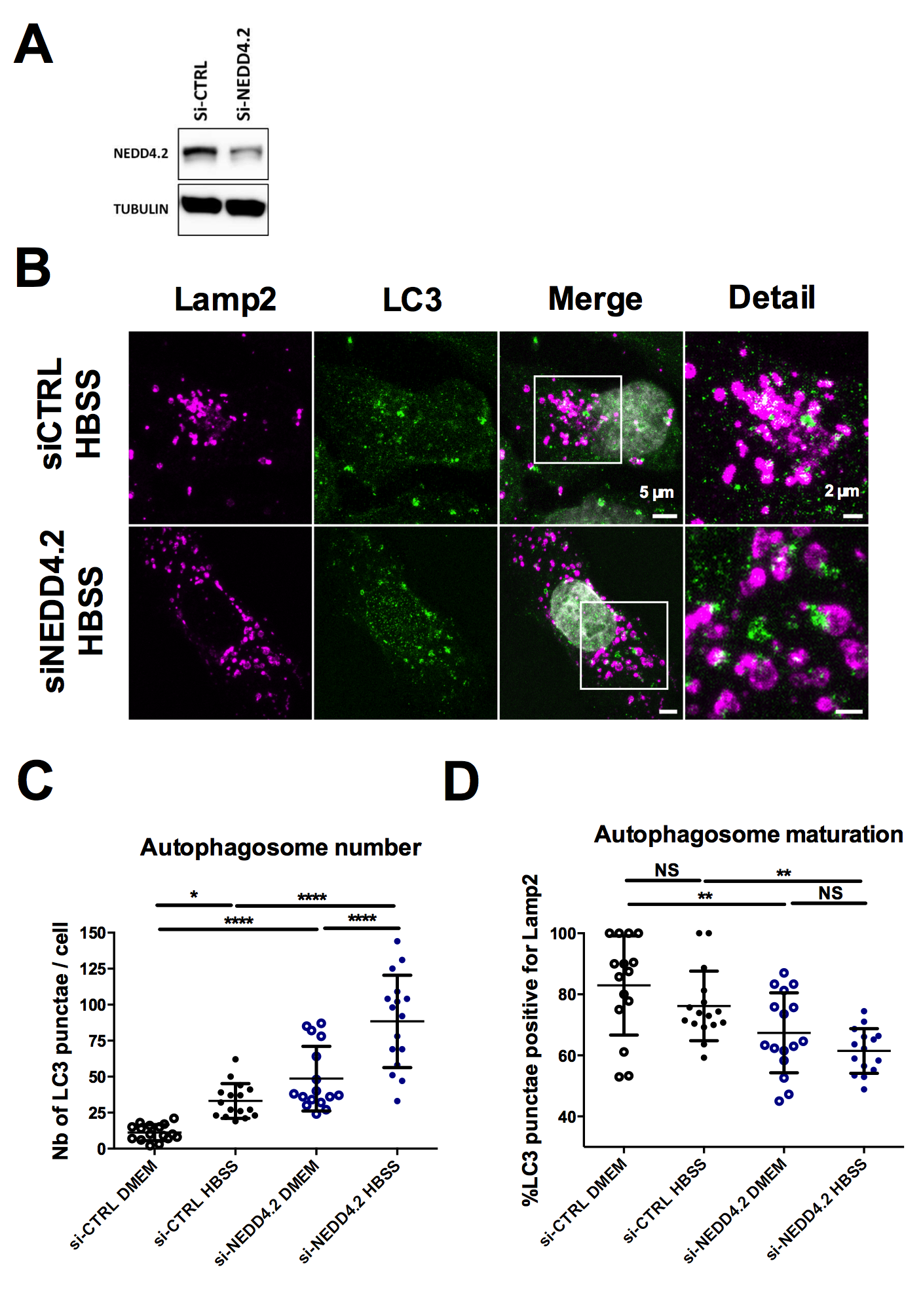

Supplement: S6 Fig — (A) Nedd4.2 expression levels were determined by western blot analysis in Nedd4.2 and control depleted cells using specific antibodies against Nedd4.2 and tubulin as loading control. (B) Representative panel of confocal images from Nedd4.2 or control depleted cells following overnight starvation in HBSS (indicated to the left) and stained with Lamp2 (magenta signal) and LC3 (green signal) specific antibodies. The detail corresponds to the boxed region. Note that autolysosomes appear white. (C) Quantification of LC3 punctae in starved vs. non-starved control cells either Nedd4.2 or control depleted (as indicated below the graph). (D) Experiment as in (C) showing the percentage of LC3 punctae also positive for Lamp2. (n>15 cells; NS: no significant; *: P<0.05; **: P<0.01; ****: P<0.0001) (TIFF) [file ppat.1006217.s006.tiff]

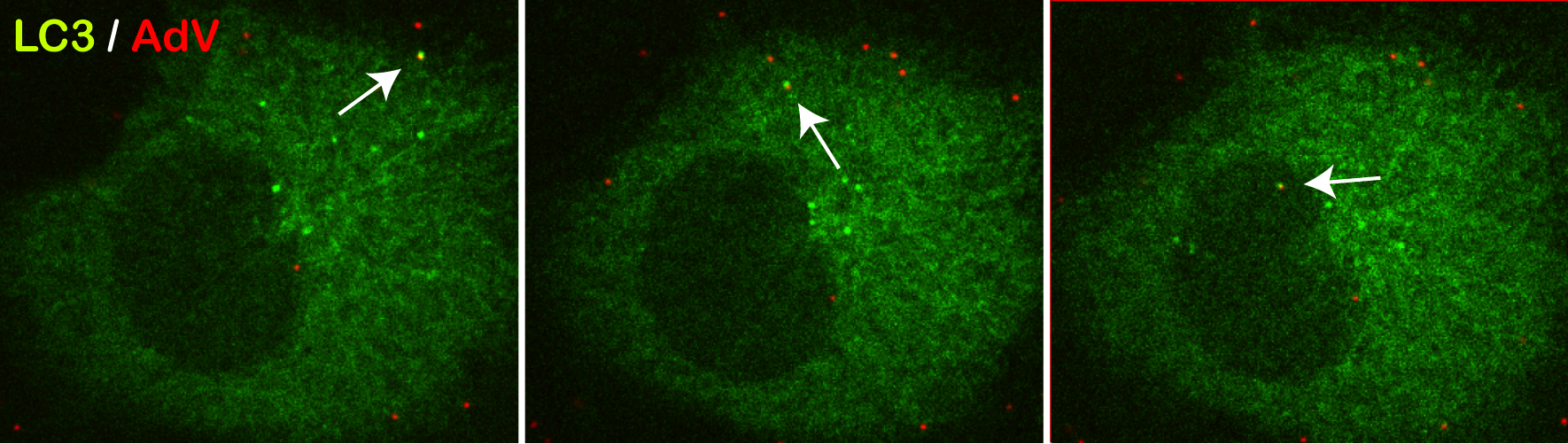

Supplement: S7 Fig — (A) Live-cell imaging showing intracellular trafficking of LC3 positive WT virus to the perinuclear region. Stable expressing U2OS-LC3-GFP cells were infected with Alexa594 coupled WT viruses and imaged using spinning-disk confocal microscopy. The images show individual frames separated by ~45 seconds from Supplemental S2 Movie. The arrow points to a virus that is LC3 positive (left panel) and moves towards the perinuclear region (middle and right panel). (TIFF) [file ppat.1006217.s007.tiff]
