# Supplementary material for: Multi-layered control of Galectin-8 mediated autophagy during adenovirus cell entry through a conserved PPxY motif in the viral capsid
Source: PLoS Pathog. 2017 Feb 13;13(2):e1006217. doi: 10.1371/journal.ppat.1006217 (PMC5325606; doi:10.1371/journal.ppat.1006217)
Supplement: S1 File — (DOCX) [file ppat.1006217.s010.docx]

### Supplemental Information

**Supplemental experimental procedures**

**Cell lines and cell culture**

U2OS cells (ATCC HTB-96^TM^), Hela cells (kindly provided by L. Gerace, Scripss research institute, La Jolla, USA) (ATCC CCL-2^TM^), MRC5 (Human lung fibroblast cell, kindly provided by J. Dechanet-Merville, CIRID, Bordeaux, France) cells (ATCC CCL-171^TM^), HepG2 cells (Hepatocarcinoma cell) (ATCC HB-8065^TM^), HuH7 (Hepatocarcinoma cell) cells (ATCC CRL-10741^TM^), MDA (Human breast adenocarcinoma cell) cells (ATCC HTB-129^TM^) (HepG2, HuH7 and MDA cells were kindly provided by F. Saltel, GREF, Bordeaux, France), HEK293 cells (ATCC CRL-1573^TM^) (kindly provided by G. Nemerow, Scripps Research Institute, La Jolla, USA) and ATG5 -/- and control MEFs (kindly provided by R. Duran, Institute Bergonie, Bordeaux, France) were grown in Dulbecco's modified Eagle's medium (DMEM) Glutamax (Invitrogen) supplemented with 10% of fetal calf serum (FCS) (Invitrogen), 100 U/mL of penicillin, 100 g/mL of streptomycin (Invitrogen) in a 5% CO_2_ atmosphere at 37°C. U2OS-Gal3-mCherry and U2OS-LC3-GFP have been constructed using flp-recombination into a stable integrated FRT site in U2OS-FRT cells [35], U2OS-FRT cells were custom made and purchased from Sirion-Biotech.

**Plasmid, siRNA and shRNA**

PX-GFP plasmid was kindly provided by Dr N. Ktistakis, Babraham institute, Cambridge UK. siRNAs were purchased as duplexes from MWG (only the reverse strand is shown): CTRL (5′-AGGUAGUGUAAUCGCCUUG), Nedd4.2 (5′-CUGUGACUUUGUGUUGUGGdTdA), [55] siGAL8#A (5′-CCCACGCCUGAAUAUUAAAGCAUUU); siGAL8#B (5′-GGACAAAUUCCAGGUGGCUGUAAAU); siGAL3#A (5′-AAGCCCAAUGCAAACAGAAUUGCUU); siGAL3#B (5′-GAGAACAACAGGAGAGUCAUUGUUU); siGAL9#A (5′-GGCUUCAGUGGAAAUGACAUUGCCU); siGAL9#B (5′-UGUGCAACACGAGGCAGAACGGAGG), [14]. Prevalidated lentiviral vectors encoding shRNAs in the vector backbone pLKO.1 against ATG5, p62 and NDP52 contains puro-resistant gene were purchased from SIGMA. Lentiviral vector production and titration was done by the service platform for lentiviral vector production of the Institut Fédératif de Recherche FR Transbiomed at Bordeaux University.

**Antibodies**

Antibodies used for western blotting in this study were: Rabbit anti-LC3 dillution 1:1000 (Cell signaling), mouse anti-GAPDH dilution 1:1000 (Sigma), serum anti-AdV 1:5000 (kindly provided by R. Iggo, Institut Bergonie, Bordeaux), rabbit anti-ATG5 dilution 1:1000 (Sigma), rabbit anti-Galectin8 1:500 (Abcam), rabbit anti-Galectin9 1:500 (Abcam) and rat anti-mCherry 1:1000 (ChromoTek). Antibodies used for immunofluorescence in this study were: anti-LC3 dillution 1:500 (Cell signaling) mouse anti-hexon 8C4 1:500 (Santa cruz), serum anti-AdV 1:500 (kindly provided by R. Iggo, Institut Bergonie, Bordeaux), mouse anti-p62 1:250 (BD Biosciences), rabbit anti-NDP52 (CALCOCO1, abcam), rabbit anti-Galectin8 1:250 (abcam), mouse and rabbit anti-pericentrin dilution 1:300 (abcam), mouse anti-PVI dilution 1:250 [10], rabbit anti-PVII dilution 1:250 (kindly provided by D. Engel), rabbit anti-Nedd4.2 dilution 1:1000 (abcam), mouse anti-Lamp2 1:250 (clone H4B4, Abcam) and secondary antibodies anti-mouse or anti-rabbit coupled with alexa488, alexa546, alexa555 or alexa647 respectively at a dilution of 1:500 (life technologies).

**Image analysis**

Confocal pictures were taken on a Leica SP5 confocal microscope equipped with Leica software. Confocal stacks where taken every 0.3 µm with a pinhole setting of 1 using threefold oversampling for all channels to achieve high local resolution. Images were processed using ImageJ. ***Quantification of single signal:*** counting of each single signal was performed using a semi-automatic macro designed on ImageJ. Stacks from confocal images where combined as Z-projection, to eliminate the background the threshold was manually defined using maximum intensity, then the number of objects was counted. For each condition a minimum of 10 cells was analyzed. ***Quantification of colocalizations:*** individual cells were imaged and analyzed using a semi-automatic macro designed on ImageJ. For every image channels were separated and stacks where combined as Z-projection then a particles analysis was performed (using maximum intensity with a manual threshold) and used to create a mask identifying objects (e.g viruses or stain for a cellular marker). Superposition of masks was done to identify colocalizations events followed by quantification. For virus colocalization analysis >50 particles were analyzed for each cells and a minimum of 10 cells is analyzed for each condition. ***Transport assay analysis:*** individual cells were imaged and analyzed using a semi-automatic macro designed on ImageJ. After channels separation and creation of Z-projection two masks were created. One contained the pericentrin signal (which is a single signal) and the other one contained the staining to be analyzed (e.g virus particles or LC3 punctae). Two concentric circles were created in ImageJ (with 10µm and 20µm in diameter) around the pericentrin signal. After superposition of both masks, virus particles or LC3 punctae were counted inside the first circle (10µm radius), in the region between the two circles (10-20µm) or outside the two circles (>20µm).
